# *IL32* downregulation lowers triglycerides and type I collagen in di-lineage human primary liver organoids

**DOI:** 10.1016/j.xcrm.2023.101352

**Published:** 2024-01-16

**Authors:** Kavitha Sasidharan, Andrea Caddeo, Oveis Jamialahmadi, Francesca Rita Noto, Melissa Tomasi, Francesco Malvestiti, Ester Ciociola, Federica Tavaglione, Rosellina M. Mancina, Alessandro Cherubini, Cristiana Bianco, Angela Mirarchi, Ville Männistö, Jussi Pihlajamäki, Vesa Kärjä, Stefania Grimaudo, Panu K. Luukkonen, Sami Qadri, Hannele Yki-Järvinen, Salvatore Petta, Silvia Manfrini, Umberto Vespasiani-Gentilucci, Vincenzo Bruni, Luca Valenti, Stefano Romeo

**Affiliations:** 1 Department of Molecular and Clinical Medicine, Institute of Medicine, The Sahlgrenska Academy, Wallenberg Laboratory, University of Gothenburg, Gothenburg, Sweden; 2 Precision Medicine Lab, Biological Resource Center Unit, Department of Transfusion Medicine, Fondazione IRCCS Ca’ Granda Ospedale Maggiore Policlinico, Milan, Italy; 3 Department of Pathophysiology and Transplantation, Università degli Studi di Milano, 20122 Milan, Italy; 4 Operative Unit of Clinical Medicine and Hepatology, Fondazione Policlinico Universitario Campus Bio-Medico, Rome, Italy; 5 Department of Medical and Surgical Sciences, University Magna Graecia, Catanzaro, Italy; 6 Department of Medicine, University of Eastern Finland and Kuopio University Hospital, Kuopio, Finland; 7 Institute of Public Health and Clinical Nutrition, University of Eastern Finland, Kuopio, Finland; 8 Clinical Nutrition and Obesity Centre, Kuopio University Hospital, Kuopio, Finland; 9 Department of Pathology, University of Eastern Finland and Kuopio University Hospital, Kuopio, Finland; 10 Section of Gastroenterology and Hepatology, PROMISE, University of Palermo, Palermo, Italy; 11 Operative Unit of Endocrinology and Diabetes, Fondazione Policlinico Universitario Campus Bio-Medico, Rome, Italy; 12 Research Unit of Clinical Medicine and Hepatology, Department of Medicine and Surgery, Università Campus Bio-Medico di Roma, Rome, Italy; 13 Department of Medicine, University of Helsinki and Helsinki University Hospital, Helsinki, Finland; 14 Minerva Foundation Institute for Medical Research, Helsinki, Finland; 15 Department of Internal Medicine, Yale University, New Haven, CT, USA; 16 Department of Cardiology, Sahlgrenska University Hospital, Gothenburg, Sweden; 17 Research Unit of Endocrinology and Diabetes, Department of Medicine and Surgery, Università Campus Bio-Medico di Roma, Rome, Italy; 18 Operative Unit of Bariatric Surgery, Fondazione Policlinico Universitario Campus Bio-Medico, Rome, Italy

**Keywords:** natural killer cell transcript 4, NK4, steatotic liver disease, SLD, fatty liver disease, FLD, downregulation, minor allele, spheroids, primary liver organoid, liver fibrosis, triglyceride, non-invasive test, NIT

## Abstract

Steatotic liver disease (SLD) prevails as the most common chronic liver disease yet lack approved treatments due to incomplete understanding of pathogenesis. Recently, elevated hepatic and circulating interleukin 32 (IL-32) levels were found in individuals with severe SLD. However, the mechanistic link between IL-32 and intracellular triglyceride metabolism remains to be elucidated. We demonstrate *in vitro* that incubation with IL-32β protein leads to an increase in intracellular triglyceride synthesis, while downregulation of *IL32* by small interfering RNA leads to lower triglyceride synthesis and secretion in organoids from human primary hepatocytes. This reduction requires the upregulation of Phospholipase A2 group IIA (*PLA2G2A*). Furthermore, downregulation of *IL32* results in lower intracellular type I collagen levels in di-lineage human primary hepatic organoids. Finally, we identify a genetic variant of *IL32* (rs76580947) associated with lower circulating IL-32 and protection against SLD measured by non-invasive tests. These data suggest that *IL32* downregulation may be beneficial against SLD.

## Highlights

• IL-32β incubation increases hepatocyte intracellular triglycerides
• Human liver organoids were composed by primary hepatocytes and hepatic stellate cells
• *IL32* downregulation lowers hepatic triglycerides and COL1A1 by upregulating *PLA2G2A*
• IL-32 rs76580947 associates with lower circulating IL-32 and ALT levels

## Graphical abstract

**Figure:**
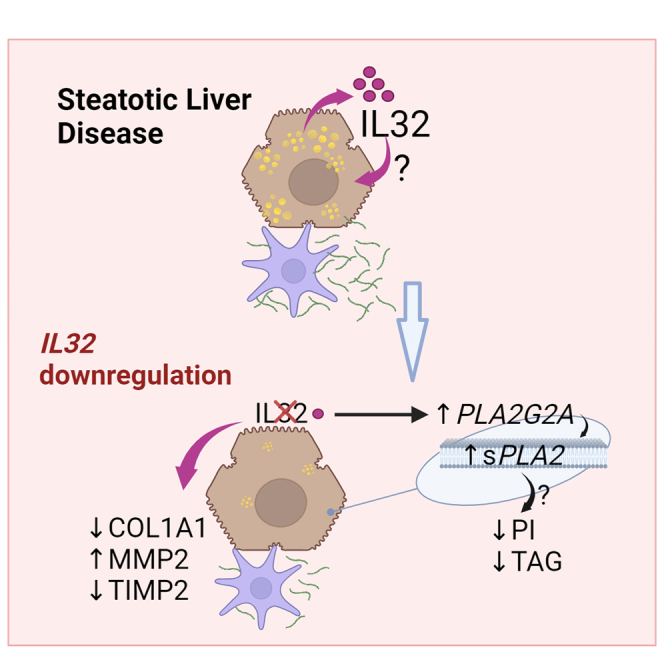

## Teaser

Elevated hepatic and circulating IL-32 levels were found in individuals with severe SLD. Sasidharan et al. show that IL-32β incubation increases intracellular triglycerides while downregulating *IL32* leads to lower triglyceride and COL1A1 accumulation in human primary liver organoids.

## Introduction

Steatotic liver disease (SLD), previously known as non-alcoholic fatty liver disease (NAFLD), is rapidly prevailing as the most common liver disease worldwide with an estimate of one-quarter of the global population affected.^1^^,^^2^ SLD, also known as metabolic-associated fatty liver disease,^3^ encompasses a wide spectrum of conditions ranging from uncomplicated liver lipid accumulation to progressive steatohepatitis with possible evolution to cirrhosis and hepatocellular carcinoma.^4^^,^^5^ Even though therapies targeting dyslipidemia and diabetes effectively decrease liver fat and improve metabolic co-morbidities,^6^ due to the heterogeneity of SLD, there are still no approved drugs for its treatment. Thus, reliable biomarkers and effective therapies are highly needed.^7^

By examining the differential hepatic gene expression of morbidly obese individuals, we and others identified interleukin 32 (IL-32) as a highly upregulated transcript in those with severe liver damage, namely metabolic dysfunction-associated steatohepatitis (MASH) significant liver fibrosis and arterial hypertension.^8^^,^^9^^,^^10^ We then demonstrated the potential of this protein as a novel circulating biomarker for detecting severe SLD non-invasively.^8^ Serendipitously, transcriptomic analyses of liver from a cohort of individuals at risk for SLD showed that IL-32 was associated with SLD progression.^11^

IL-32 is a cytokine first identified in natural killer and lymphocyte T cells from humans.^12^ IL-32 does not share sequence homology with the classical cytokines. Nonetheless, it is classified as a pro-inflammatory cytokine due to its ability to induce tumor necrosis factor α, IL-8, IL-6, and IL-1β.^13^ Recent evidence suggests that IL-32 is involved in inflammatory pathways regulation acting both on membrane receptors following its secretion and on intracellular signaling.^14^^,^^15^ However, several aspects related to the function of this cytokine including a putative role in lipid metabolism regulation^16^^,^^17^^,^^18^ remain unknown. IL-32 is transcribed in nine isoforms, generated by alternative splicing.^14^^,^^19^ Among these, IL-32β is the most highly expressed in the liver and correlates with the severity of SLD.^8^ The *IL32* gene is identified only in mammals. Indeed, *IL32* is completely absent in rodents, which has represented a challenge for investigating its role in *in vivo* experimental models.^20^

In the present study, we aimed to elucidate the impact of IL-32 on hepatic lipid metabolism in hepatocytes. To this end, we demonstrated a role of IL-32 in the induction of triglyceride and collagen1A synthesis and accumulation by using hepatic spheroids from immortalized and primary human cells. Consistently, we identified a genetic variant in *IL32* (rs76580947) that reduces the circulating levels of this cytokine and protect against liver steatosis in three independent study cohorts.

## Results

### IL-32 manipulation governs intra-hepatocellular lipid content in immortalized cell lines and 3D spheroids

IL-32 isoform β is the most highly expressed in the liver of individuals with SLD, while IL-32 isoforms α and γ have a relatively lower expression.^8^ To understand the effect on intracellular lipid metabolism in hepatocytes, we incubated HepG2 cells with increasing amounts (0–100 nM) of IL-32 α, β, or γ isoforms for 48 h.

**Figure 1:**
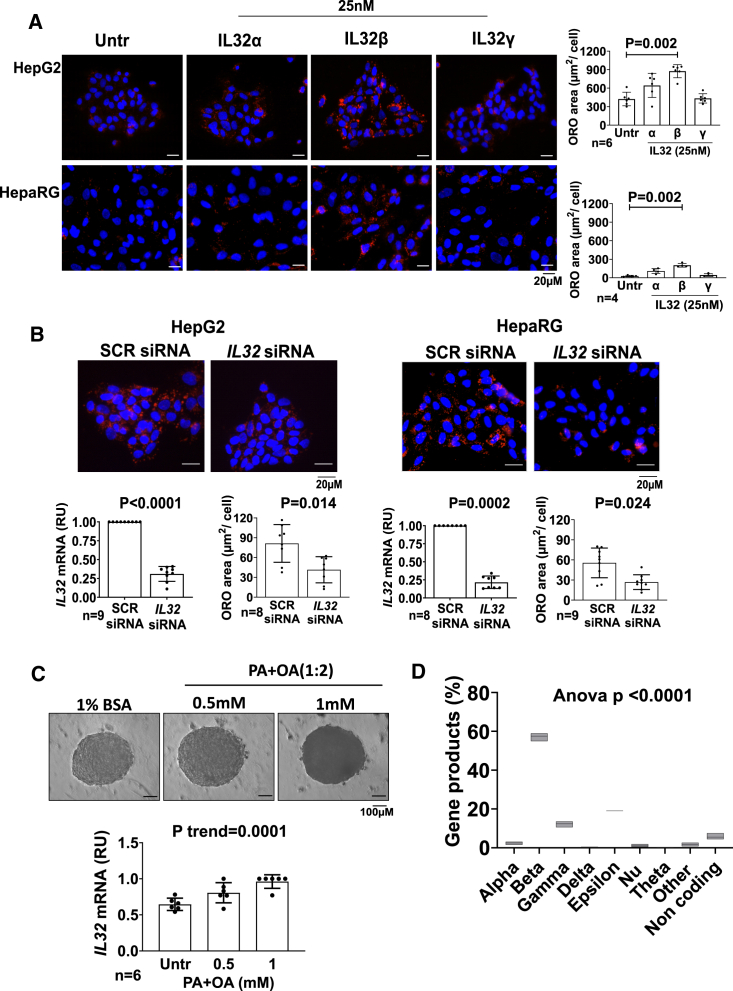
IL-32β is the most dominant isoform in HepG2+LX-2 spheroids that increases neutral fat content, and *IL32* downregulation lowers neutral fat in 2D cultured hepatocytes (A) To test the effect of IL-32 administration on intracellular fat content, immortalized human hepatic cell lines HepG2 and HepaRG were cultured in 2D and incubated with human recombinant IL-32α, IL-32β or IL-32γ isoform for 48 h. Then, intracellular neutral fat content was visualized by Oil Red O staining (ORO). ORO area quantified per DAPI stained nuclei by ImageJ, showed increased intracellular neutral fat content in both (A) HepG2 (top) and HepaRG (bottom). (B) To test the effect of *IL32* downregulation on intracellular fat content, 24 h after seeding, cells were transfected with scramble or *IL32* siRNA and grown in regular medium without FBS (HepG2) or medium supplemented with 25 μM oleic acid (HepaRG) for an additional 48 h. The average of gene knockdown efficiency was ∼70%–75% as evaluated by real-time qPCR analyzed by the 2^−ΔΔCt^ method. Intracellular neutral fat content was visualized by Oil Red O staining (ORO). ORO area quantified per DAPI stained nuclei by ImageJ showed lower intracellular neutral fat content in both HepG2 (left) and HepaRG (right). Two-sided p values were calculated by the Mann-Whitney non-parametric t test. Data shown as mean ± SD in all groups for the reported number of experiments. (C) HepG2+LX-2 cells were cultured as spheroids for 96 h exposing them to medium supplemented with 1% BSA or to increasing concentrations of a mixture of fatty acids (PA + OA, 1:2). *IL32* gene expression measured after exposure, demonstrated higher *IL32* mRNA levels with increasing intra-spheroidal triglyceride levels. The p value was calculated by test for linear trend. Data shown as mean ± SD for the reported number of experiments. (D) Percentage of *IL32* gene products versus other IL-32 isoforms was measured from RNA sequencing (RNA-seq) data and IL-32β is the most dominantly expressed *IL32* isoform in our spheroid model. The p value was calculated by one-way ANOVA. OA, oleic acid; PA, palmitic acid; RU, relative unit.

IL-32β showed the strongest effect in increasing intracellular triglycerides, roughly by 2-fold, measured by AdipoRed assay followed by IL-32α, while IL-32γ showed no effect (Figure S1). As IL-32β at a concentration of 25 nM showed the maximum effect on triglycerides accumulation, we chose this dose for the subsequent experiments. Next, we incubated HepG2 and HepaRG cells with 25 nM of IL-32 α, β, and γ for 48 h and measured the intracellular neutral fat content by Oil Red O staining (Figure 1A). Consistently, IL-32β resulted in the highest levels of intracellular lipids content in both cell lines, suggesting that IL-32 promotes neutral lipid accumulation. To confirm that IL-32 is directly involved in intra-hepatocyte triglycerides handling, we downregulated *IL32* by small interfering RNA (siRNA) and observed an approximately 50% decrease in the intracellular neutral lipid content in both HepG2 and HepaRG cell lines exposed to oleic acid (Figure 1B).

On incubation of spheroids composed of human hepatoma cells (HepG2) and immortalized hepatic stellate cells (LX2) with increasing amounts of a mixture of 1:2 palmitic and oleic acid (0–1 mM), we observed a dose dependent increase in *IL32* expression (Figure 1C). On measuring the IL-32 isoforms expression in these spheroids, IL-32β was the most expressed isoform (Figure 1D). These data confirmed the human data correlating IL-32β expression and hepatic fat accumulation and lipotoxicity.

**Figure 2:**
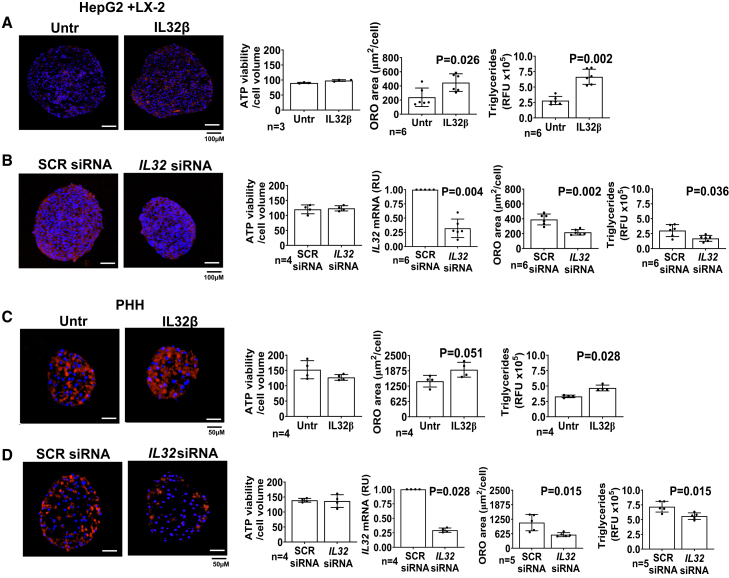
Incubation with human recombinant IL-32β increases while *IL32* downregulation lowers intracellular triglyceride content in spheroids from immortalized and human primary hepatocytes (A) HepG2+LX-2 cells were cultured as spheroids for a total of 96 h. Initially, 48 h after seeding cells the media was supplemented with 25 nM IL-32β for 48 h. (B) HepG2+LX-2 spheroids were generated by seeding cells along with negative control (SCR) siRNA or 30 nM *IL32* siRNA transfection mix for downregulation for total of 96 h. (C) Primary human hepatocytes (PHH) were cultured as spheroids for a total of 7 days. Initially, 48 h after seeding the media was supplemented with 25 nM IL-32β for an additional 5 days with media replacement every 48 h. (D) PHH were cultured as spheroids along with negative control (SCR) siRNA or 30 nM *IL32* siRNA transfection mix for downregulation, for a total of 7 days. For both spheroid models, cellular ATP levels (marker of viability) remained stable between the experimental groups. The average of gene knockdown efficiency was ∼70%–75% as evaluated by real-time qPCR analyzed by the 2^−ΔΔCt^ method, relative to beta-actin. Intracellular neutral fat content measured by Oil Red O staining and AdipoRed assay showed an increase in triglycerides content after incubation with IL-32β while *IL32* downregulation lowers triglyceride levels. Two-sided p values were calculated by Mann-Whitney non-parametric t test. Data shown as mean ± SD in all groups for the reported number of experiments. RFU, relative fluorescence unit; RU, relative unit (to beta-actin); SCR, scramble siRNA; untr, untreated.

To establish this observed effect of IL-32 β, we replicated the same experiments as the 2D cultured hepatocytes by incubating HepG2+LX-2 spheroids with 25 nM IL-32β and obtained a 2-fold increase in triglycerides and neutral lipid content (Figure 2A). Conversely, downregulation of *IL32* by using siRNA decreased by approximately 50% neutral lipid accumulation (Figure 2B).

### *IL32* downregulation lowers intracellular triglycerides and type I collagen levels in primary hepatocyte spheroids

Following our observation in immortalized spheroids, we tested incubation with human recombinant IL-32β in spheroids generated from cryopreserved primary human hepatocytes and it resulted in a higher neutral lipid content (Figure 2C), while downregulation resulted in la ower neutral lipid content (Figure 2D).

**Figure 3:**
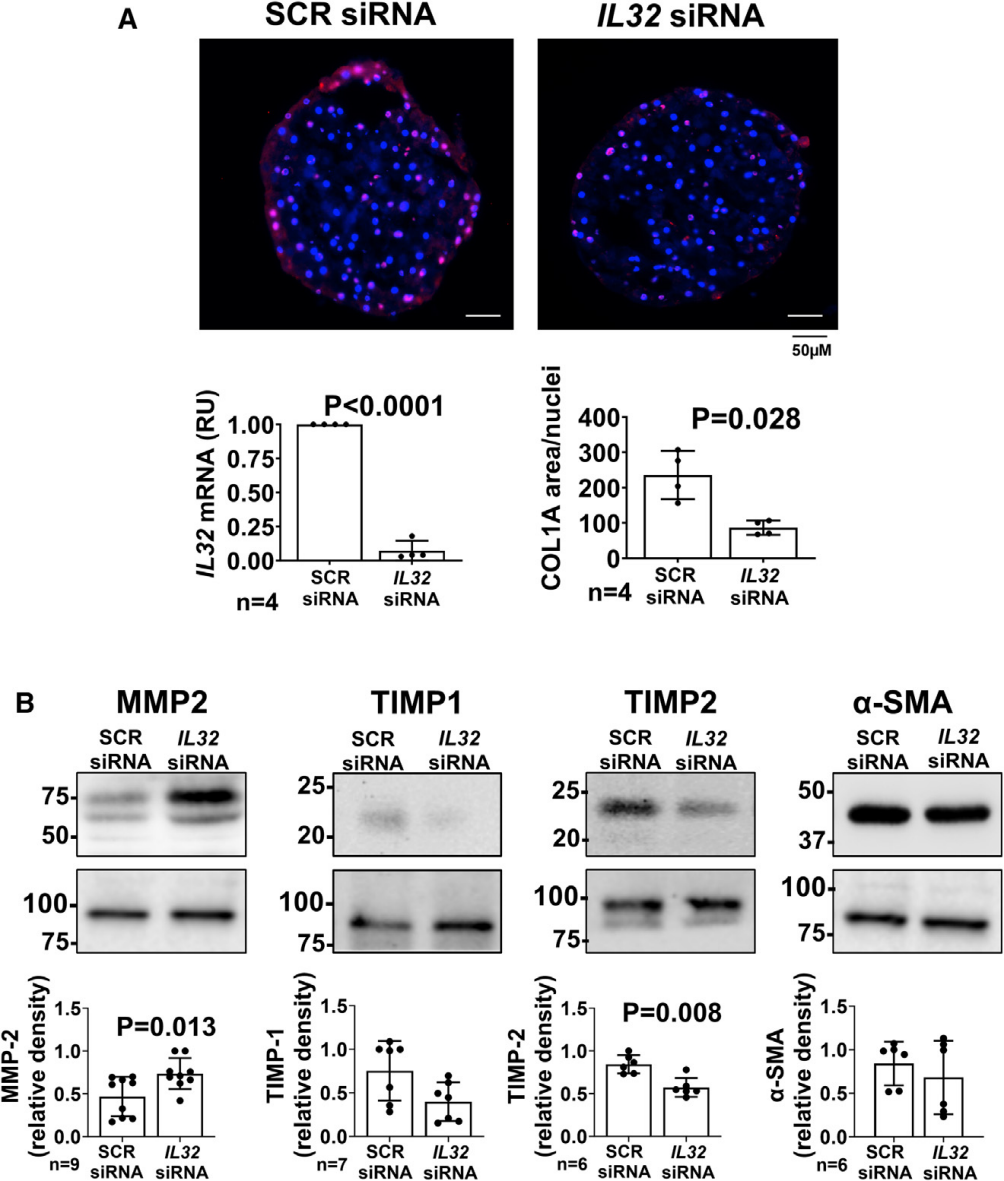
*IL32* downregulation lowers intracellular COL1A1, increases MMP2 levels, and lowers TIMP2 in primary di-lineage human spheroids Primary human hepatocytes and primary hepatic stellate cells, at the ratio 24:1, were seeded with negative control scramble (SCR) and *IL32* siRNA, at 5,000 cells/well in ultra-low attachment 96-well U-bottom ultra-low attachment plates. Fifty percent of the total media was replenished with fresh media every 48 h. (A) After 7 days of formation, spheroids were collected and 8-μM sections were subjected to immunofluorescent staining for COL1A1. Immunofluorescence was quantified by ImageJ, normalized to number of DAPI stained nuclei. The knockdown efficiency was measured by real-time qPCR, relative to beta-actin. (B) MMP2, TIMP-1, TIMP-2, and α-SMA protein levels were measured by western blotting in the cell lysate. Calnexin was used as loading control. Representative images of protein levels are shown. For each panel, data shown as mean ± SD of the reported independent experiments. Two-sided p values were calculated by Mann-Whitney non-parametric test. COL1A1, collagen Iα1; MMP2, matrix metallopeptidase 2; TIMP1, tissue inhibitor of metalloproteinase 1; TIMP2, tissue inhibitor of metalloproteinase 2.

Progression of SLD comprises liver fibrosis worsening. To test whether *IL32* downregulation has a potential effect on ameliorating fibrosis, we measured collagen protein levels by immunofluorescent staining of type I collagen (COL1A1) in spheroids composed of a 24:1 mixture of cryopreserved primary hepatocytes and hepatic stellate cells from human donors. *IL32* downregulation resulted in lower COL1A1 (Figure 3A). To investigate the mechanisms underlying lower COL1A1 deposition, we examined intracellular protein levels of metalloproteinases (MMP2 and 9), tissue inhibitors of MMPs (TIMP1 and 2) and smooth muscle actin α (α-SMA). *IL32* downregulation resulted in higher MMP2, lower MMP9, and TIMP2; there were no changes in TIPM1 and α-SMA (Figure 3B).

### IL-32 downregulation lowers, and IL-32 β increases, intracellular triglycerides synthesis

**Figure 4:**
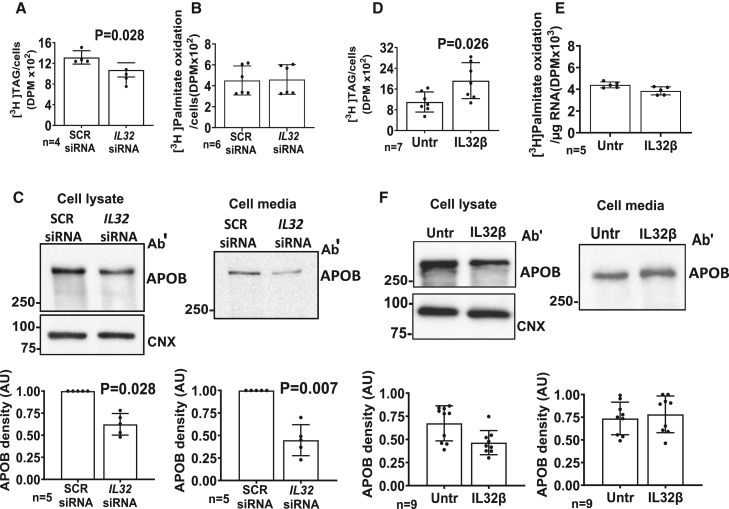
Endogenous *IL32* downregulation lowers, and incubation with recombinant IL-32 β increases, intracellular triglycerides synthesis For endogenous *IL32* downregulation experiments, HepG2+LX-2 spheroids were generated by seeding cells along with negative control (SCR) siRNA or *IL32* siRNA transfection mix for a total of 96 h. For IL-32β incubation experiments, initially, 48 h after seeding HepG2+LX2 cells (24:1), the media was supplemented with 25 nM IL-32β for another 48 h. In both conditions, newly synthesized triglycerides were separated by TLC and quantified by scintillation counting after incubation with 6 μCi/mL ^3^H-glycerol plus 1.5 mM glycerol for 12 h. (A) Reduction in *de novo* triglyceride synthesis after IL-32 downregulation. (D) Increase in *de novo* triglyceride synthesis after 25 nM IL-32β incubation. Cells were incubated with 8.5 μCi/mL ^3^H-palmitate +55 μM/L palmitic acid for 6 h, after which palmitate was precipitated with BSA and perchloric acid and quantified by scintillation counting. (B and E) Graph shows no difference in beta oxidation in both experimental groups. APOB-100 synthesis and secretion levels were measured by immunoblotting. (C) Decrease in APOB-100 in cell lysate and cell culture supernatant after IL-32 downregulation. (F) No changes in APOB-100 levels in cell lysate and culture medium, after incubation with 25 nM IL-32β. The reported number of experiments were performed independently. Representative blots are presented. For all experiments, two-sided p value was calculated by Mann-Whitney non-parametric t test. Data shown as mean ± SD. AU, arbitrary units; DPM, disintegrations per minute; TAG, triacylglycerol.

Intracellular hepatocyte triglyceride levels are a function of intracellular triglyceride synthesis, utilization via beta oxidation and secretion via apolipoprotein B (APOB). To understand the mechanisms underlying the effect of *IL32* downregulation on lipid metabolism, we examined the impact on lipid metabolism pathways in HepG2+LX-2 spheroids. More specifically, to study the effect on triglyceride synthesis, after *IL32* downregulation by siRNA for 96 h, spheroids were incubated with ^3^H-glycerol for 12 h. Then, thin-layer chromatography was performed to measure the newly synthesized triglycerides (Figure 4A). We found that *IL32* downregulation resulted in a reduction of the newly synthesized triglycerides in our spheroid model.

Next, to measure fatty acids utilization, after *IL32* downregulation for 96 h, spheroids were incubated with ^3^H palmitate for 6 h. We found no differences in the soluble fraction radioactivity of the media after *IL32* downregulation, indicating no changes in fatty acid utilization by beta oxidation (Figure 4B). Then, to evaluate the effect on fatty acid secretion, we measured APOB levels in both spheroid lysates and in the culture media by immunoblotting. We observed a reduction of APOB synthesis (in the lysates) and secretion (in the cell supernatant) after *IL32* downregulation as compared with control conditions (Figure 4C).

Finally, using radiolabeled ^3^H-glycerol, we also demonstrated vice versa that incubation with 25 nM IL-32β resulted in an increase in the synthesis of triglycerides (Figure 4D). However, no changes were observed in fatty acid utilization (Figure 4E) or APOB protein levels in the cell lysate and culture medium (Figure 4F).

### Phospholipase A2 group IIA drives IL-32-mediated reduction in triglyceride synthesis

To understand how *IL32* downregulation governs triglyceride synthesis we examined differentially expressed genes by RNA sequencing in HepG2+LX-2 spheroids after *IL32* downregulation. We found that 1,422 genes were downregulated and 1,354 were upregulated when comparing *IL32* siRNA versus negative control siRNA-treated spheroids.

**Figure 5:**
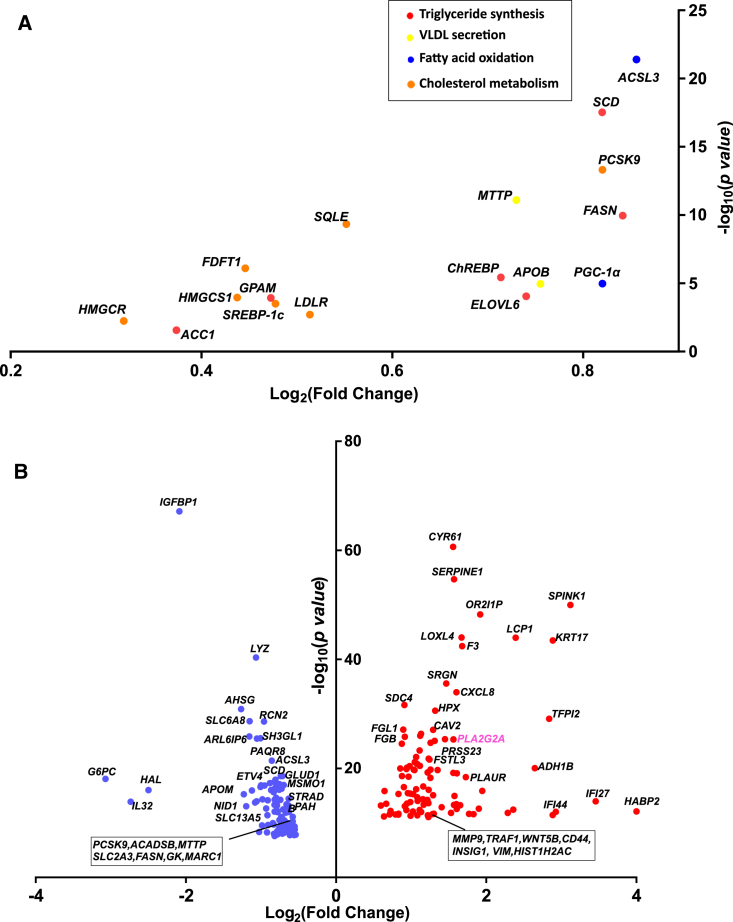
Differentially expressed genes reveals downregulation of key genes for lipid metabolism in HepG2+LX2 spheroids (A) Key genes of lipid metabolism differentially expressed in spheroids after *IL32* downregulation as compared with scramble. (B) Top 100 differentially expressed genes after *IL32* downregulation as compared with scramble. Data are presented as log2-fold change in expression and –log_10_ of p values adjusted using the Benjamini and Hochberg’s approach for controlling the false discovery rate (FDR). VLDL, very low-density lipoprotein.

Downregulation of *IL32* reduced expression levels of SREPB-1c (*SREBF1*), the master regulation of triglyceride synthesis and other key genes like *GPAM*, *ACC*, and *FASN*. Consistent with protein levels, there was a decrease in *APOB* mRNA and microsomal triglyceride transfer protein (*MTTP)*. These data are consistent with the experiments done in the same settings with the radiolabeled tracers showing a reduction in triglycerides synthesis. Finally, there was also a reduction in genes involved in fatty acid (PGC-1α [*PPARGC1A*] and *ACSL3*) oxidation (Figure 5A). However, no difference in the experiments with radiolabeled tracers was seen in the same experimental setting.

When we examined the top 100 differentially upregulated genes (Figure 5B) we identified *PLA2G2A*, a secretory phospholipase. *PLA2G2A* encodes for secretory calcium-dependent phospholipase A2 group IIA, which hydrolyzes the ester bond of the fatty acyl group of phospholipids,^21^^,^^22^ and has previously been implicated in antimicrobial defense^23^^,^^24^ and inflammatory response.^25^^,^^26^ Secretory phospholipase A2 has also been found to have an impact on insulin sensitivity and metabolism.^27^^,^^28^

**Figure 6:**
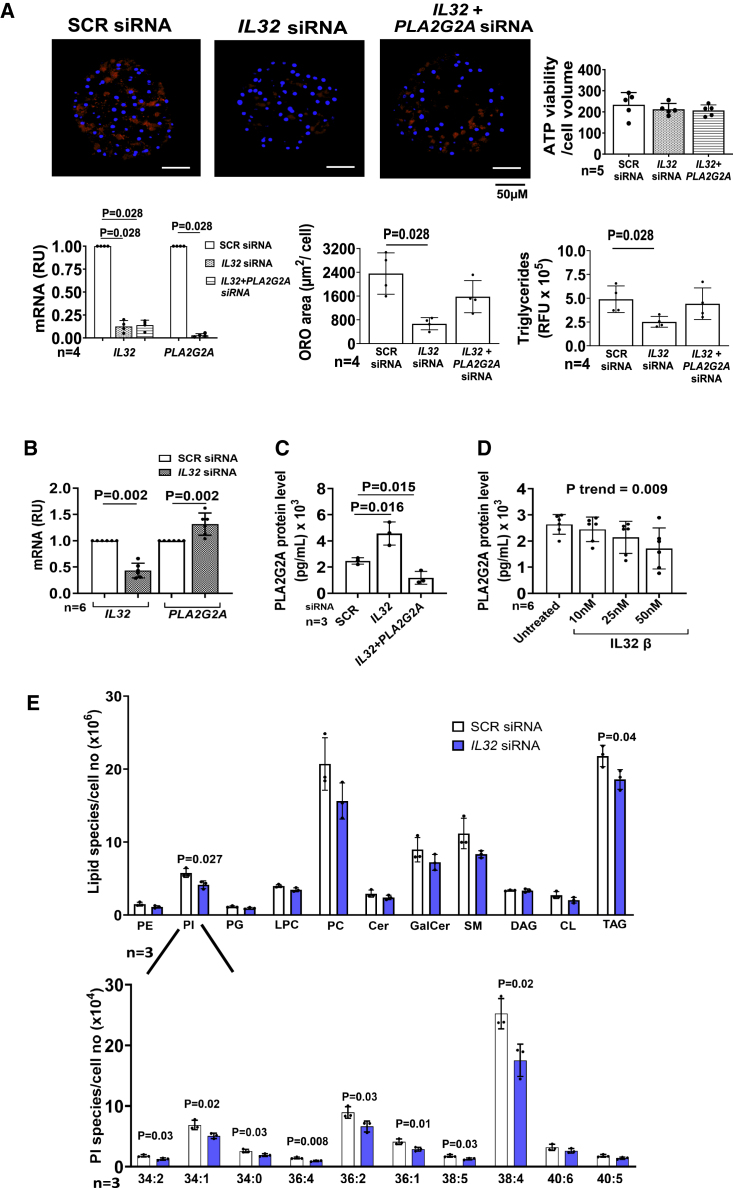
Co-downregulation of *PLA2G2A* and *IL32* abolished the IL-32-mediated intracellular triglycerides lowering and *IL32* downregulation reduces intracellular PI levels in human primary spheroids Primary human hepatocytes were cultured as spheroids and incubated with negative control (SCR) siRNA or 30 nM *IL32*, and combination of *IL32* and *PLA2G2A* for a total of 7 days. (A) Cellular ATP levels (marker of viability) were not different within the three groups. There was an 80%^–90% reduction in mRNA levels of *IL32* and *PLA2G2A*, relative to beta-actin. Intracellular neutral lipid content (measured by Oil Red O staining) normalized to nuclei (stained by DAPI) were lower after *IL32* downregulation while the co-downregulation of *PLA2G2A* and *IL32* abolished this reduction. (B) *IL32* downregulation results in higher *PLA2G2A* mRNA levels measured by real-time PCR. (C) *IL32* and *PLA2G2A* were downregulated individually or in combination in primary hepatocyte spheroids and PLA2G2A levels were measured by human PLA2G2A ELISA in the culture medium. PLA2G2A levels were higher after *IL32* downregulation and lower after co-downregulation of *IL32* and *PLA2G2A*. (D) After 2 days from seeding, hepatocyte spheroids were incubated with 10, 25, and 50 nM human recombinant IL-32β for 5 days. PLA2G2A was measured from cell culture supernatant using human PLA2G2A ELISA and we observed a dose dependent decrease in secreted PLA2G2A levels with increasing concentration of IL-32β. The p values were calculated by test for linear trend. (E) Lipid fingerprint measured by liquid chromatography- quadrupole time-of-flight-mass spectrometry demonstrated lower total PI and triglycerides (top) levels after *IL32* downregulation. There was a reduction in all PI species, except 40:5 and 40:6, with the largest effect size in 38:4 (bottom). For each part, data are shown as mean ± SD of the reported independent experiments. Two-sided p values calculated with unpaired t test for n = 3 and Mann-Whitney non-parametric t test for n > 3. Cer, ceramides; CL, cardiolipins; DAG, di-acylglycerides; GalCer, galactosyl ceramides; LPC, lysophosphatidylcholine; ORO, Oil Red O; PE, phosphatidylethanolamine; PG, phosphatidylglycerol; RFU, relative fluorescence units; RU, relative units; PC, phosphatidylcholine; SM, sphingomyelin; TAG, triacylglycerols.

To elucidate whether the upregulation of this phospholipase was implicated in the mechanism underlying the decrease in accumulated triglycerides, we downregulated *IL32* and *PLA2G2A* in HepG2+LX-2 spheroids. More specifically, spheroids were generated with only downregulation of *IL32* or with co-downregulation of *IL32* and *PLA2G2A*. As expected, downregulation of *IL32* resulted in a decrease in the intracellular triglyceride content as measured by both Oil Red O staining and AdipoRed assay. However, this effect was abolished by the co-downregulation of *IL32* and *PLA2G2A* (Figure S3). These experimental conditions were replicated using cryopreserved primary hepatocytes spheroids and virtually identical results were obtained (Figure 6A). In these spheroids, the downregulation of *IL32* led to an increase in the mRNA levels of *PLA2G2A* as measured by real-time PCR, consistent to what we observed in the transcriptome profile of HepG2+LX2 spheroids (Figure 6B). Furthermore, in these spheroids, we also detected an increase in secreted PLA2G2A levels from the cell supernatant, measured using human PLA2G2A ELISA kit (Figure 6C). Conversely, the incubation with increasing doses of human recombinant IL-32β (10–50 nM) resulted in a dose-dependent decrease in secreted levels of PLA2G2A (Figure 6D). Next, we investigated the lipidomic fingerprint associated with *IL32* downregulation by liquid chromatography- quadrupole time-of-flight-mass spectrometry (Figure 6E). Consistent with Oil Red O and AdipoRed staining, we observed a reduction in total triglycerides. Moreover, there was a reduction in total phosphatidylinositol (PI) and all species, except for PI 40:6 and 40:5, with the largest effect size in 38:4. Taken these together, these experimental models suggest that the mechanism behind the *IL32*-mediated decrease in intracellular triglycerides requires intact expression of *PLA2G2A.*

### *IL32* rs76580947 variant associates with protection against liver disease and lower IL-32 circulating levels

**Figure 7:**
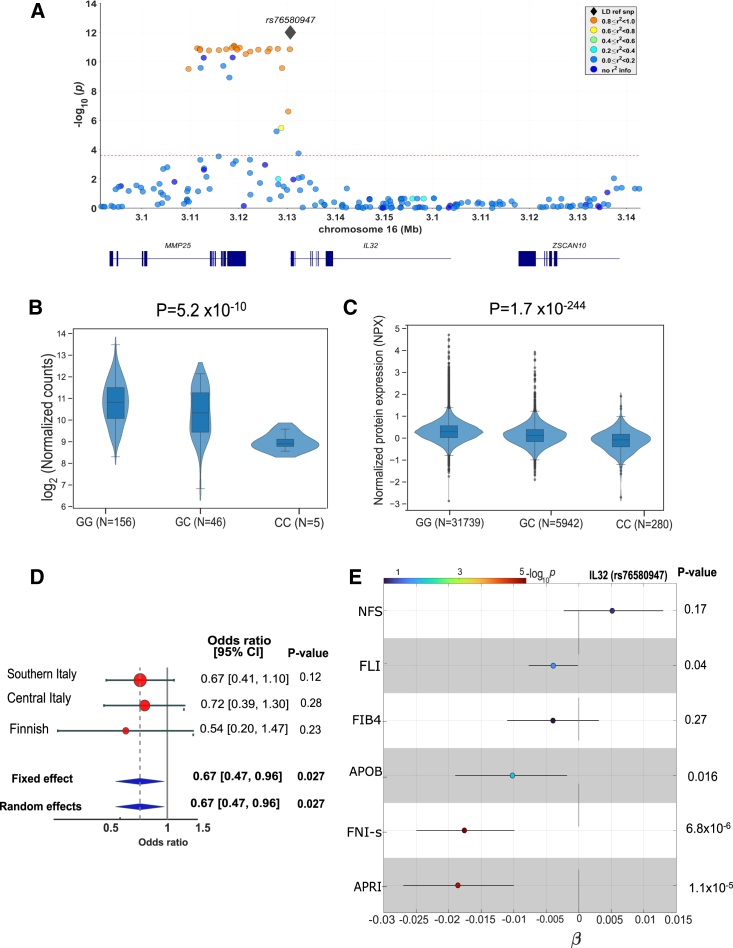
IL-32 rs76580947 minor allele associates with lower IL-32 expression, severe liver steatosis, and lower liver non-invasive tests (A) Regional plots of association between *IL32* common genetic variants (minor allele frequency of >0.01) and ALT in the European subset of UK biobank. The x axis shows the variant positions (GRCh37); the y axis shows the –log10 p values. The gray diamond represents rs76580947, with the strongest association in the plotted region (IL-32 ± 50 Kbp), for which its pairwise LD with other variants is color coded as shown on the figure. (B) The association between rs76580947 and hepatic IL-32 mRNA levels was tested in 207 individuals from the MAFALDA cohort adjusting for age, gender, percentage of coding bases (a quality control measure from the Picard toolkit), RNA Integrity Number (RIN), and five surrogate variables detected by surrogate variable analysis.^32^ Carriers of the variant have lower IL-32 mRNA levels. Data shown as violin plots and adjusted p values are reported. (C) The association between *IL32* rs76580947 stratified by genotype and IL-32 plasma protein level in 365,495 European participants from UK Biobank was tested using a linear regression analysis adjusted for age, gender, body mass index, first 10 genomic principal components, and array batch. Violin plot shows the normalized Protein eXpression (NPX) values that were rank-based inverse normal transformed prior to the analysis. (D) Forest plot of association and meta-analysis for *IL32* rs76580947 with steatosis in three independent cohorts: Southern Italy (N = 425), Central Italy (N = 245), and Finnish (N = 745). The plot shows protection against severe liver steatosis (steatosis absence or mild vs. severe; fixed-effect p = 0.027). The association was tested by a binary logistic regression analysis under an additive genetic model adjusted by age, gender, body mass index, and recruitment center (only for the Finnish cohort). Pooled effect estimates were calculated using inverse-variance-weighted fixed effects meta-analysis. (E) The association between *IL32* rs76580947 and clinical liver fibrosis scores and APOB levels in 365,495 European participants from UK Biobank. The analysis was performed under an additive model, using linear regression adjusting for age, gender, body mass index, the first 10 genomic principal components, and array batch. All traits were rank-based inverse normal transformed prior to the analysis. CI, confidence interval.

To translate our findings in humans, and to gain insight into causality of the previously reported association between gene expression and SLD severity, we examined the relationship between *IL32* genetic variations and liver damage in individuals from the UK Biobank. Specifically, we selected all common genetic variants (minor allele frequency of >0.01) within ±50 Kbp flanking regions at the *IL32* locus on chromosome 16 (n = 194) and tested the association with alanine transaminase (ALT) level, a marker of liver damage associated with fatty liver, in a total of 425,671 European participants from the UK Biobank (Figure 7A).^29^ The regional plot shows a block of genetic variants in high linkage disequilibrium (LD) with a strong association even after adjusting for Bonferroni correction. The risk haplotype was tagged by rs76580947, which was the most significant hit. This variant remained the index variant within the locus (a margin of 1 Mbp) after LD clumping, and the independence of this variant was further confirmed by an approximate stepwise model selection procedure as implemented in genome-wide complex trait analysis-conditional and joint association analysis.^30^^,^^31^

Next, we examined the phenotype associated with rs76580947 minor allele and found that carriers of the variant had lower ALT (beta = −0.02, p = 9.9 × 10^−13^) and that they also consistently showed lower aspartate aminotransferase (AST) (beta = −0.02, p = 3.2 × 10^−10^), reduced risk of severe liver disease (adjusted odds ratio, 0.82; 95% confidence interval, 0.71–0.96; p = 0.013), and lower total cholesterol (beta = −0.01; p = 0.009) (Table S1). However, no difference was found in liver fat content levels measured by proton density fat fraction in the UK Biobank (beta = −0.002; p = 0.86). No difference in diabetes mellitus was observed among rs76580947 carriers.

Next, we tested the association between rs76580947 and hepatic *IL32* mRNA levels in 207 individuals from the Molecular Architecture of FAtty Liver Disease in individuals with obesity undergoing bAriatric surgery (MAFALDA) cohort (from central Italy) and we found that carriers of the variant have lower *IL32* mRNA levels (Figure 7B). Further, we also examined the association between IL-32 rs76580947 minor allele and 1,463 plasma protein levels from 365,495 European individuals from the UK Biobank. The carriers of the variant have substantially lower circulating IL-32 levels (beta = −0.04; p = 1.8 × 10-^244^) (Figure 7C and Table S3) indicating the *IL32* variant associates with lower gene expression.

Furthermore, we wanted to test if the rs76580947 variant was associated with protection against liver steatosis. We examined a total of 770 individuals at risk for liver disease (with morbid obesity), with Italian (South Italy, N = 425; Central Italy [MAFALDA], N = 245) and Finnish (N = 745) ancestry from the Liver Biopsy Cohort, in whom measurement of hepatic steatosis by liver biopsy was available. Meta-analyses of the association between the rs76580947 and severe hepatic steatosis (steatosis absence or mild vs. severe) showed a reduction in severe hepatic steatosis prevalence in carriers of the variant (Figure 7D). No differences were found in liver inflammation, ballooning, and severe fibrosis (absence and mild vs. severe fibrosis) (Table S4). Also, we examined the impact of rs76580947 minor allele on IL-32 circulating levels in a total of 955 Italian individuals with dysmetabolism from the Liver-BIBLE 2020 cohort.^10^^,^^33^ The clinical features of Liver-BIBLE 2020 cohort stratified according to rs76580947 genotype are shown in Table S2. The frequency of *IL32* rs76580947 G>C minor allele was 0.119 in agreement with gnomAD database, and the genotype distribution conformed to Hardy-Weinberg equilibrium (p = 0.28). Carriage of the minor C allele was associated with a lower frequency of elevated IL-32 circulating levels, defined as levels in the top quartile of its distribution (Table S2 and Figure S2) (adjusted odds ratio, 0.66; 95% confidence interval, 0.46–0.94; p = 0.02).

Finally, we assessed the association of rs76580947 minor allele with the following fibrosis non-invasive tests in the UK Biobank: Fibrosis 4 index (FIB4),^34^ Fatty liver index (FLI),^35^ NAFLD fibrosis score (NFS),^36^ AST to platelet ratio index (APRI),^37^ and fibrotic NASH index (FNI).^38^ Interestingly, carriers of the variant showed lower FLI (beta = −0.018; p = 0.04 marker of steatosis), FNI (beta = –0.017; p = 6.8 × 10^−6^, a marker of fibrotic MASH), and APRI (beta = −0.019; p = 1.2 × 10^−5^, a marker of fibrosis), respectively. However, we did not find any association with FIB4 and NFS. Finally, the variant was also associated with lower circulating APOB levels (Figure 7E and Table S5). Finally, we examined the association between the variant and diagnosis of SLD from summary statistics of four large independent studies^39^^,^^40^^,^^41^ (Figure S4). However, we did not see any association between the variant and SLD. There are at least two reasons that may explain this result: (1) The effect size of the reduction on ALT levels of the variant is small (beta = 0.02) and, therefore, this may not translate in a clinical appreciable difference, (2) the definition of SLD of these studies is done with *International Classification of Diseases* codes and it is very underestimated, and (3) these studies are mostly from general population.

Taken all this together, these data are consistent with the *in vitro* experiments that decreasing IL-32 level may be beneficial against liver steatosis and fibrosis in individuals at risk for SLD.

## Discussion

The main findings of this study are that (1) downregulation of *IL32* reduces the intracellular triglycerides and type collagen I levels in primary human liver spheroids by decreasing triglyceride synthesis, and (2) a common *IL32* genetic variant minor allele associates with lower *IL32* hepatic mRNA and circulating protein in humans, resulting in protection against SLD.

In previous studies, IL-32 was highly upregulated in the liver of individuals with severe liver disease and steatosis due to SLD or hepatitis C virus infection.^8^^,^^11^^,^^42^ We also showed that IL-32 is detectable in the circulation and correlates with hepatic fat and liver damage.^8^ Therefore, in the present study we sought to perform molecular studies to understand the relationship between IL-32 and SLD. We performed *in vitro* studies in 2D and 3D with immortalized cells showing that downregulation of *IL32* resulted in lower intracellular triglycerides content, while incubation with IL-32 resulted in an increase in this lipid species. Results were confirmed in spheroids composed of cryopreserved primary hepatocyte and cryopreserved primary hepatic stellate cells from human donors. Radioactive tracer studies showed that the increase in neutral lipid content was due to heightened triglyceride synthesis with no changes in intracellular triglycerides utilization. In this model, we also found a reduction in the synthesized and secreted APOB. The amount of APOB secreted in humans is a function of the amount of triglycerides present in hepatocytes.^43^ Therefore, it is likely that the observed reduction in APOB synthesis and secretion may be reactive to the overall reduction in hepatocyte triglycerides content. Interestingly, downregulation of *IL32* resulted in lower intracellular PI levels. We have previously shown that changes in the hepatic PI remodeling due to lower expression of *MBOAT7* increases liver triglyceride content.^44^^,^^45^ The results of this work reinforce the notion of a role of PI in intrahepatic triglycerides homeostasis.

To gather insights on the mechanisms underlying the beneficial effect of *IL32* downregulation, we performed transcriptomic analyses in HepG2+LX2 spheroids. In line with the experiments with radioactive tracers, among the most downregulated genes, there were many related to triglycerides synthesis, whereas among the most upregulated genes we found phospholipase A2 group IIA (*PLA2G2A*). *PLA2G2A* is a secreted enzyme highly expressed in the liver^46^ and involved in glycerolipid remodeling.^28^^,^^47^^,^^48^ Consistent with our data, a previous study shows that overexpression of human PLA2G2A results in a decrease in genes involved in hepatic triglyceride synthesis and protects against hepatic steatosis in mice fed a high-fat diet.^27^ Co-downregulation of *IL32* and *PLA2G2A* abolished the reduction in intracellular lipids mediated by *IL32* downregulation, indicating that this effect required PLA2G2A. Lipidomic data from primary human spheroids showed that, after *IL32* downregulation, the largest effect size is in reduced PI 38:4. This is consistent with higher levels of PLA2G2A releasing arachidonic acid from PI. However, it is unclear what is the exact mechanism linking IL-32 and PLA2G2A with intracellular triglycerides synthesis.

Liver fibrosis is the major determinant of liver related events in individuals with chronic liver disease. Therefore, we sought to test the effect of *IL32* downregulation on COL1A1 levels in spheroids generated with cryopreserved human primary hepatocyte and hepatic stellate cells. Downregulation of *IL32* resulted in lower intracellular COL1A1 levels. We have previously shown that liver triglyceride excess causes liver fibrosis and severe liver disease.^7^^,^^49^ Therefore, we speculate that this effect may be mediated by the primary reduction in spheroid lipid content. However, we cannot exclude a direct effect of *IL32* downregulation on liver fibrosis.

To reinforce the role of IL-32 in human chronic liver disease, we examined a total of 194 genetic variants in the gene and flanking regions for association with ALT levels in individuals from the UK Biobank. We identified a strong association between rs76580947 and lower ALT. To examine the association between the sequence variant and liver disease phenotypes we used noninvasive tests. The rs76580947 minor allele was associated with lower severity of SLD as estimated by the FLI, fibrotic MASH by the FNI, and fibrosis by the APRI. However, the variant was not associated with liver triglyceride content in the overall UK Biobank. Interestingly, in line with our *in vitro* studies in spheroids, carriers of the variant had also lower circulating levels of circulating APOB-containing lipoproteins. Importantly the minor allele was associated with a robust reduction in IL-32 circulating levels in Europeans from the UKBB and in approximately 900 individuals from Italy.

Next, to confirm the association between the variant and liver disease, we genotyped a total of 1,415 individuals from Italy and Finland from the Liver Biopsy Cohort (with available liver biopsy), and showed that carriers of the variant had less severe steatosis. We then examined mRNA levels in a subset of this cohort (MAFALDA, n = 207) and observed that the minor allele was robustly associated with a reduction in *IL32* mRNA levels in the liver. These results are consistent with the findings in the UK Biobank. Collectively, the data support the notion that *IL32* downregulation is beneficial against SLD.

In conclusion, we show that downregulation of *IL32* reduces triglyceride synthesis resulting in lower intracellular lipid content in classical 2D culture and human liver spheroids. Consistently, a common *IL32* genetic variant associates with lower indices of liver damage and lower hepatic steatosis in humans. These results highlight IL-32 as a potential therapeutic target to treat SLD.

### Limitations of the study

Our study does not include *in vivo* murine models. However, IL-32 is not present in rodents, restricting the use of these models to test the effect of IL-32 downregulation. Moreover, we provide evidence in humans of a genetic variation robustly associated with lower transaminase levels and with lower hepatic steatosis. IL-32 is a secreted protein and, at this point, it is not clear if the reduction in intracellular triglyceride content is mediated by a receptor-mediated extracellular effect or an intracellular effect. Even though we identified PLA2G2A as a potential mediator of the IL-32 effect, further studies are warranted to pin down the exact molecular mechanisms underlying the reduction in intracellular triglyceride content due to *IL32* downregulation.

## STAR★Methods

### Key resources table

**Table:** 

REAGENT or RESOURCE	SOURCE	IDENTIFIER
**Antibodies**		

anti-APOB	Santa cruz biotechnology	sc13538;RRID:AB_626690
anti-CALNEXIN	Sigma-Aldrich	4731;RRID:AB_476845
Alexa Fluor™594 donkey anti-rabbit IgG (H + L)	Thermo fisher scientific	A21207;RRID:AB_141637
Anti-Mouse IgG horseradish peroxidase (HRP) conjugated secondary antibody	Abcam	NA931V;RRID:AB_772210
Anti-Rabbit IgG horseradish peroxidase (HRP) conjugated secondary antibody	Abcam	NA934;RRID:AB_772206
anti-COL1A1	Sigma-Aldrich	HPA011795;RRID:AB_1847088
anti-MMP2	Abcam	ab92536;RRID:AB_10561597
anti-TIMP1	Abcam	ab76003;RRID:AB_1310463
anti-TIMP2	Abcam	ab180630;RRID: AB_3076748
anti-α-SMA	Abcam	ab5694;RRID:AB_2223021

**Chemicals, peptides, and recombinant proteins**		

3^H^-Glycerol	Perkin elmer	NET022L001MC
Acetic Acid	Sigma-Aldrich	1603051000
Bis-Tris	Applichem	A1025
Bovine Serum Albumin	Sigma-Aldrich	A8806
Chloroform	Sigma-Aldrich	650498
Diethyl ether	Sigma-Aldrich	673811
D-Mannitol	Sigma-Aldrich	M4125
EDTA 0.5M	Thermo fisher scientific	AM9261
Fetal Bovine Serum	Corning	35-079-CV
Glycerol	Sigma-Aldrich	G9012
H_2_ SO4	Sigma-aldrich	339741
IL32α	R&D systems,	3040-IL
IL32β	R&D systems,	6769-IL
IL32γ	R&D systems,	4690-IL/CF
L-glutamine	Cytiva	SH30034.01
Methanol	Sigma-Aldrich	439193
M-PER protein extraction reagent	Thermo fisher scientific	VS0152
NaCl	Sigma-Aldrich	S9625
Non essential amino acids	Cytiva	SH30238.01
Nonfat dried milk powered	Panreac applichem, itw reagents	78505
Oil Red O	Sigma-Aldrich	O0625
Oleic Acid	Sigma-Aldrich	O1383
Palmitic Acid	Sigma-Aldrich	P-0500
Palmitic acid, (9,10-3H(N))	Perkin elmer	NET043005MC
Paraformaldehyde	Sigma-Aldrich	16005
PBS (+calcium, +magnesium)	Cytiva	SH30264.01
PBS (-calcium, - magnesium)	Cytiva	SH30028.02
Penicillin-Streptomycin	Cytiva	SV30010
Petroleum ether	Sigma-Aldrich	101316-46-5
Scintillation fluid	Perkin elmer	6013329
Sodium Carbonate	Sigma-Aldrich	S7795
Sodium Chloride	Sigma-Aldrich	S3014
Sodium dodecyl sulfate	VWR	444464T
Sodium Pyruvate	Cytiva	SH30239.01
Sucrose	Sigma-Aldrich	S9378
Tris base	Sigma-Aldrich	252859
Triton X-100	Merck millipore	108603.1000
Trypan Blue stain 0.4%	Invitrogen	T10282
Trypsin	Cytiva	SH30236.01

**Critical commercial assays**		

AdipoRed™ Assay Reagent	Lonza	PT-7009
Cell-Titer-Glo® Luminescent Cell Viability	Promega	G7571
ECL substrate (Immobilon Western Chemiluminescent HRP Substrate	Merck millipore	WBKLS0500
High Capacity cDNA Reverse Transcription Kit	Thermo fisher scientific	4368813
Human IL32 DuoSet ELISA kit	R&D system	DY3040
Human PLA2G2A kit	Invitrogen	EH369RB
Lipofectamine™ 3000 Transfection Reagent	Thermo fisher scientific	L3000001
RNAeasy Plus Mini kit	Qiagen	74104
TaqMan Gene Expression Master Mix	Thermo fisher scientific	4369016

**Deposited data**		

HepG2-LX2 Spheroid RNA-seq	NCBI-SRA	PRJNA1035504
Raw data, unprocessed western blots, unprocessed fluorescence images and summary statistics	Mendeley data	10.17632/sx47w852k5.1

**Experimental models: cell lines**		

HepG2 hepatoma cells	ATCC	HB-8065
HepaRG	Thermo fisher scientific	HPRGC10
LX-2	ATCC	SCC064
Cryopreserved primary human hepatocytes- donor 1	BioIVT	BGW-M00995-P
Cryopreserved primary human hepatocytes- donor 2	BioIVT	BGF-M00995-P
Cryopreserved primary human hepatic stellate cells- donor 1	BioIVT	TFE-S00354
Cryopreserved primary human hepatic stellate cells- donor 2	BioIVT	NGU-S00354

**Oligonucleotides**		

Taqman gene expression assay- ACTB	Thermo fisher scientific	Hs01060665_g1
Taqman gene expression assay- IL 32	Thermo fisher scientific	Hs00992441_m1
*IL32* siRNA mix 1:1:1	Thermo fisher scientific	s17656, s17657, s17658
TaqMan® SNP Genotyping Assays IL32 rs76580947	Thermo fisher scientific	C_102039340_10
Taqman gene expression assay- IL 32	Thermo fisher scientific	Hs00179898_m1
*PLA2G2A* siRNA mix 1:1:1	Thermo fisher scientific	s10589, s10591, s224271
Scramble siRNA	Thermo fisher scientific	AM4611

**Software and algorithms**		

R v4.2.2	R project	https://www.r-project.org/
BOLT-LMM v2.4.1	Loh et al., 2015^50^	https://alkesgroup.broadinstitute.org/BOLT-LMM/BOLT-LMM_manual.html
CFX Manager software v.3.1	Bio-rad	N/A
DESeq2 v.1.38.3	Love et al.,2014^51^	RRID:SCR_015687
FastQC	Babraham bioinformatics	https://www.bioinformatics.babraham.ac.uk/projects/fastqc/
GraphPad Prism	Graphpad software	version 9 v.1.52h, NIH
Image Lab software v.6.1	Bio-rad	N/A
ImageJ v1.38.3		https://ImageJ.net/ij/
RSEM v1.3.3	Li et al., 2011^52^	N/A
STAR v2.7.10a	Dobin et al., 2013^53^	https://github.com/alexdobin/STAR
sva v3.46.0	Leek et al., 2012^32^	RRID:SCR_012836
Trimmomatic v0.39	Bolger et al., 2014^54^	http://www.usadellab.org/cms/index.php?page=trimmomatic

**Other**		

DAPI	Sigma-aldrich	D9542
Countess cell counting chamber slides	Invitrogen	C10283
Countess II Automated cell counter	Invitrogen	AMQAX1000
Fluorescence Mounting Medium	Dako	S3023
Nucleocassette™	Chemometec	941–0001
Nucleocounter	Eppendorf	M1293
OCT cryomount	Histolab	SH30264.01
Vivaspin® 500 spin columns	Sartorius	VS0152
QIA Symphony	Qiagen	9001297

### Resource availability

#### Lead contact

Further information and requests for resources and reagents should be directed to and will be fulfilled by the lead contact, Stefano Romeo (Stefano.romeo@wlab.gu.se).

#### Materials availability

This study did not generate new unique reagents.

#### Data and code availability

For UK Biobank, all individual-level phenotype/genotype data are accessible via a formal application to the UK Biobank http://www.ukbiobank.ac.uk. Owing to study participants’ privacy and data protection, the RNA-seq data of the Liver Biopsy Cohort, central Italy (MAFALDA) and Liver-BIBLE cohorts can only be made available on request to the corresponding authors for collaborative projects. Bulk RNA-seq data of the HepG2-LX2 immortalized cell line spheroids are deposited in the NCBI SRA under the BioProject identifier PRJNA1035504. Any additional information required to reanalyze the data reported in this work paper is available from the lead contact upon request.

### Experimental model and study participant details

#### Cell lines and primary cell culture

HepG2 hepatoma cells (ATCC, Menassas, VA, USA) were grown in MEM supplemented with 10% FBS and HepaRG (terminally differentiated HepaRG cells, Thermo Fisher Scientific) cells were grown in William’s media supplemented with 10% FBS. HepG2 + LX-2 spheroids were generated as previously described.^55^ In detail, HepG2 cells and LX-2 (ATCC) cells were trypsinised from T25 flasks and counted using nucleocassettes (Chemometec). They were seeded at a 24:1 ratio into 96-well round-bottomed ultra-low attachment plates (Corning) at 2000 cells/well in MEM supplemented with 10% FBS. They were incubated at 37°C in a humidified atmosphere of 5% CO2 and grown for a total of 96h. There is no need for centrifugation or change of medium for these immortalized cell line derived spheroids. For the primary human spheroids in Figures 2 and 3, cryopreserved primary human hepatocytes and primary human hepatic stellate cells from a commercial provider, BioreclamationIVT (BioIVT) were used. All donor characteristics of primary cells used are described in Table S6. The vials were thawed at 37°C and 1 vial of cells was added to 5mL of prewarmed BioIVT provided CP medium (CP media contains serum). Next, cell viability and count were measured using Trypan blue exclusion method on countess 3, automated cell chamber. Cells were seeded at 5000 viable cells per well onto 100μL of serum containing CP medium. Spheroids that were composed of primary human hepatocytes and primary human hepatic stellate cells, were seeded at the ratio 24:1 on 96-well round-bottomed ultra-low attachment plates (Corning). Plates were centrifuged at 100 g × 5 min. Once the cells collect at the bottom, self-aggregation causes formation of spheroids. At day 1 after seeding, 100μL of BioIVT serum free maintenance medium (HI medium) was added to make a total of 200μL per well. Next, every 48h, 50% of media was replenished for fresh serum free HI medium until day 7. Spheroid viability was determined using CellTiter-Glo Luminescent Cell Viability Assay kit (Promega, Madison, WI, USA). Cellular ATP was normalized to spheroid volume. Images of spheroids were taken by an Axio Vert.A1 inverted microscope (Carl Zeiss AG). Spheroids composed by only primary human hepatocytes for the experimental setup described in Figure 6A, were similarly generated as detailed above.

#### Human participants

We identified an *IL32* gene variant (rs76580947) associated with lower ALT levels in the UK Biobank (UKBB). The UKBB is a large-scale biomedical database with in depth data from >500,000 participants (including baseline assessment, physical measures and genetic data) recruited between 2006 and 2010 and aged 40–69 years. The UKBB received ethical approval from the National Research Ethics Service Committee North West Multi-Centre Haydock (ref. 16/NW/0274).^56^ Data used in this study were obtained under application number 37142. The European subset of UKBB was defined by adding the participants (N = 425,671) who self-reported as being “Irish” or “any other White background” (after removal of outliers based on first 6 genetic principal components) to the subset of White British ancestry, and further excluding the individuals with more than 10 putative third-degree relatives, with a mismatch between their self-reported and genetically inferred gender, having putative sex chromosome aneuploidy, who had withdrawn consent, and were identified by the UKBB as outliers based on heterozygosity and missingness. The phenotype was then validated in four different human liver biopsy study cohorts comprised of individuals with the diagnosis of non-alcoholic steatohepatitis based on the presence of steatosis with lobular necroinflammation and ballooning or fibrosis.^29^ Study cohorts used are: a) southern Italy who are 466 individuals from the Gastrointestinal and Liver Unit of the Palermo University Hospital, Palermo, Italy as described previously^29 29^ b) Finland, comprising 512 individuals from the Northern Savo Hospital District, Kuopio, Finland,^57^ and 312 from the Hospital District of Helsinki and Uusimaa, Finland^58^ and c) Central Italy comprising of 245 individuals from the “Molecular Architecture of FAtty Liver Disease in individuals with obesity undergoing bAriatric surgery (MAFALDA)”.^59^ Briefly, the central Europe cohort (MAFALDA), consists of consecutive individuals with morbid obesity eligible for bariatric surgery, without clinical history of alcohol abuse (men, ≥30 g/d; women, ≥20 g/d), viral hepatitis, and other causes of liver disease recruited at Campus Bio-Medico University, Rome, Italy. At the preoperative assessment visit, clinical, anthropometric, and laboratory data were collected using standardized procedures. On the day of surgery, a laparoscopic-guided percutaneous liver core biopsy was obtained and scored according to NAFLD activity score (NAS) classification.^60^ MASH diagnosis was established by the pathological assessment based on Brunt et al. criteria with at least grade one for steatosis, ballooning, and lobular inflammation.^61^ The MAFALDA study has been approved by the Local Research Ethics Committee (no. 16/20) and it was conducted in accordance with the principles of the Declaration of Helsinki. All participants gave written informed consent to the study. Histological evaluation of liver disease has been performed by experienced pathologists blinded to patients’ clinical data. All study cohort participants were genotyped for the variant rs76580947 by TaqMan assay (ThermoFisher Scientific). All genotypes were performed in duplicate with 100% concordance rate. Chronic liver disease and cirrhosis^29^ were defined according to International Classification of Diseases, 10th edition (ICD-10) as previously described in detail. Severe liver disease was defined as described before by merging ICD-10 codes C22.0, I85.0, I85.9, K70.3, K70.4, K72.1, K72.9, K74.1, K74.2, K74.6, K76.6, K76.7, Z94.4 in any of in-hospital admissions, death or cancer registries (data-fields 41270, 40001, 40002, and 40006). Individuals were excluded if they were diagnosed with other causes of liver disease, chronic viral hepatitis (B18, B19, E83.0, E83.1, K71, K74.3, K74.4, K74.5, K75.2, K75.3, K75.4, K75.8, K75.9) or any other type of cancer except C22.0.^62^ Lastly, the effect of the IL32 genetic variant on circulating IL32 levels was validated in the Liver-BIBLE cohort 2020 comprising of 955 healthy individuals with dysmetabolism presented for blood donation from June 2019 to February 2021 at the Transfusion Medicine and Hematology unit of Fondazione Ca’ Granda Hospital (Liver-Bible cohort 2020).^10^^,^^33^ Briefly, inclusion criteria were age 40–65, associated with at least three of the following features: overweight or obesity (Body mass index (BMI) > 25 kg/m^2^), increased fasting glucose or type 2 diabetes (fasting glucose≥100 mg/dl), triglycerides≥150 mg/dl, HDL<45/55 in M/F, arterial hypertension. Individuals with chronic degenerative disorders, except for well controlled arterial hypertension, compensated hypothyroidism, and type 2 diabetes not requiring pharmacological therapy, were excluded from the cohort at first evaluation. The clinical features of the cohort are presented in Table S2. DNA was extracted from peripheral blood collected at the time of enrollment by the QIA Symphony (Qiagen). Participants were genotyped for the variant rs76580947 by TaqMan assay (ThermoFisher Scientific). All genotypes were performed in duplicate with 100% concordance rate. For genetic studies, no randomization was performed for individuals.

### Method details

#### Treatment with recombinant IL32

After 24 h of seeding, HepG2 and HepaRG cells were exposed to human recombinant IL32α (3040-IL; R&D Systems), IL32β (6769-IL; R&D Systems) or IL32γ (4690-IL/CF; R&D Systems) for 48 h. For HepG2+LX-2 spheroids, 48 h after seeding 25 ng/mL IL32β was supplemented along with 100μL fresh medium for an additional 48 h to make a total of 200μL. For PHH spheroids, 25 ng/mL IL32β was supplemented after 24 h of seeding along with fresh medium. Fifty per cent of total media was replenished with fresh media every 48 h. With every media change IL32β was also supplemented to maintain a final concentration of 25 ng/mL in each well.

#### Measurement of viability

Total cellular adenosine triphosphate (ATP) was measured by the Cell-Titer-Glo cell viability assay (Promega) according to manufacturer’s instructions with slight modifications. Briefly, single spheroids were transferred into a white 96-well assay plate (Corning) with 50 μL PBS. Then, 50 μL of assay reagent were added and vigorously mixed to allow the reagent to penetrate the spheroid, after which the plates were incubated in the dark for 25 min at room temperature. Luminescence was measured in a spectrophotometer (SpectraMax i3; Molecular Devices Inc.). Cellular ATP was normalized to spheroid volume. Images of spheroids were taken by an Axio Vert.A1 inverted microscope (Carl Zeiss AG).

#### In vitro-siRNA transfection and gene expression

For downregulation experiments, 30nM of negative control scramble (SCR siRNA) (AM4611, Thermo fisher scientific), *IL32* siRNA (mixt of s17656, s17657 and s17658 (1:1:1, total 30nM), Thermo Fisher scientific) or *PLA2G2A* siRNA (mix of s10589, s10591 and s224271, s17658 (1:1:1, total 20nM) Thermo Fisher Scientific) was transfected with Lipofectamine 3000, as per manufacturer’s instructions. For 2D culture, transfection was performed 24 h after seeding cells and cells were transfected for 48 h till endpoint analyses. For spheroids, transfection mix was supplemented in the media at the time of seeding to facilitate maximum uptake of siRNA. HepG2+ LX-2 spheroids were grown for 96 h till harvesting endpoint, while PHH and PHH + PHHSC spheroids were left to form for 7 days until endpoint analysis. Even with media changes, no additional transfections were performed. RNA from cells and spheroids were extracted with the RNeasy Plus Mini Kit (Qiagen) and reverse transcribed using high-capacity cDNA reverse transcription kit (Thermo Fisher Scientific) according to the manufacturer’s instructions. Gene expression was assessed by Real-Time qPCR using master mix (Life Technologies) and TaqMan probes for *IL32* and *PLA2G2A* according to the manufacturer’s protocol. All reactions were performed in triplicate. Data were analyzed using the 2^−ΔΔCt^ method normalized to beta actin.

#### Quantification of intracellular fat content

Intracellular neutral fat content was visualized by Oil Red O (ORO, Sigma-Aldrich Inc., St. Louis, MO, USA) staining as described before.^63^ HepG2 cells in 2D culture, were not treated with additional fatty acids prior to ORO staining. HepaRG cells were incubated with regular growth media supplemented with 25μM Oleic acid (OA) conjugated to BSA. Oil Red O stock was prepared by dissolving 0.5 g of Oil Red O in 100mL 100% isopropanol. Working solution was prepared by mixing 6 parts of stock and 4 parts water. Cells in 2D, were grown in glass coverslips for ORO staining. They were exposed to 20% isopropanol for 30 s, followed by ORO staining for 20 min, washing with double distilled water and lastly with DAPI for nuclei staining. For experiments on spheroids with fatty acid incubation, palmitic acid (PA) and oleic acid (OA) (sigma Aldrich) at a ratio of 1:2 was dried under nitrogen gas and resuspended in BSA containing media and left to properly mix overnight at 37°C. Two different concentrations of 0.5mM and 1mM were prepared, along with medium supplemented with 1% BSA that served as control. HepG2+LX-2 spheroids were generated in this medium for a total of 96 h. Spheroids were fixed with 10% w/v paraformaldehyde (Sigma-Aldrich) for 2 h, then incubated with 20% sucrose in phosphate buffered saline (PBS) overnight, after which they were washed with PBS and embedded with OCT cryomount (Histolab, Västra Frölunda, Sweden). Then, 8-μm-thick sections were made using a cryostat (Leica, Wetzlar, Germany) and transferred onto glass slides after which ORO staining was performed as described above. Nuclei was stained with DAPI. Images were acquired using an Axio KS 400 Imaging System and AxioVision 4.8 software (Zeiss) at 40× magnification. The ORO-stained area was normalized to the number of DAPI- stained nuclei and quantified using an in-house macro in ImageJ (v.1.52h, NIH). The AdipoRed lipid assay to quantify triglycerides in spheroids was performed as previously described.^55^ Briefly, single spheroids were moved onto a new 96-well clear bottom plate with 200μL PBS. 20μL trypsin was added and incubated at 37°C for 20 min. Next, 7 μL of AdipoRed reagent were added, mixed well with a multichannel pipette to disrupt the spheroids, and incubated for 10 min. Fluorescence was measured with excitation at 485 nm and emission at 572 nm in the SpectraMax i3 (Molecular Devices Inc.).

#### Radiolabeled tracer studies

For measurement of *de novo* triglyceride synthesis, HepG2+LX-2 spheroids were generated with transfection of negative control SCR siRNA or *IL32* siRNA for 84 h. Then, they were incubated with MEM without supplemented FBS but including 6 μCi/mL 3H-glycerol (PerkinElmer), 1.5 mM glycerol (Sigma-Aldrich) and 1% BSA (Sigma-Aldrich) for 12 h. Spheroids were pooled and washed with PBS to remove media contaminants. Lipids were extracted by the Folch extraction procedure. Briefly, 300 μL of 2:1 chloroform:methanol (v/v) (Sigma-Aldrich) and 100 μL of acidified solution (17mM NaCl, 1mM H2 SO4) (Sigma-Aldrich) was added to the collected spheroids and sonicated (Soniprep 150) for 10 s to disrupt them. Samples were centrifuged at 15,000g for 10 min, the organic phase was collected and dried under nitrogen gas. Lipids were reconstituted by resolubilizing in 50μL chloroform and separated on TLC silica plates (Merck-Millipore). Triolein (Sigma-Aldrich) was used a marker for triglycerides and Petroleum ether: diethyl ether: acetic acid (40:60:1, v/v) was used a mobile phase. The spots corresponding to triglycerides were visualized with iodine vapor and were cut and added to vials with scintillation fluid (PerkinElmer). The newly synthesized triglycerides were measured with a scintillation counter (Beckman Coulter LS6500) as disintegrations per minute (DPM). Equal number of spheroids similar to one replicate of the sample was pooled and trypsinised for cell counting. Data were normalized for the number of cells in spheroids counted with nucleocounter (M1293; Eppendorf). For measurement of fatty acid oxidation, HepG2+LX-2 spheroids were generated with transfection of negative control SCR siRNA or IL32 siRNA for 90 h. Then, they were incubated with MEM without supplemented FBS but with 8.5 μCi/mL 3H-palmitate (PerkinElmer); 55 μM palmitic acid (Sigma-Aldrich), and 1% BSA for 6 h. Thereafter, 500 μL of pooled media was collected and the radiolabeled palmitate was precipitated by adding 50 μL of 20% BSA and 27 μL of 70% perchloric acid. The supernatant was collected after centrifugation at 12,000 rpm for 5 min, after which a second aliquot of 20% BSA was added. This was repeated for a total of three times. Then, the final supernatant was added to vials with scintillation fluid. Radioactivity was measured in a scintillation counter as disintegrations per minute (DPM). The data were normalized for the number of cells in the spheroids.

#### Immunoblot analysis

Spheroid lysates were prepared by pooling spheroids from same group, into M-PER protein extraction reagent (ThermoFisher Scientific) supplemented with 10% protease inhibitor (SIGMAFAST, Sigma-Aldrich Co.) and briefly homogenising. The supernatant was collected and protein concentration estimated for immunoblotting. For immunoblotting of spheroid cell media, 500μL of spheroid supernatant was collected and concentrated 10-fold using Vivaspin 500 spin columns (Sartorius Stedim Lab Ltd, UK). Proteins were separated by SDS-PAGE and transferred to nitrocellulose membranes in accordance with standard procedures. For the detection of APOB, which has a molecular weight of approximately 500kDa, the proteins were separated on 6% PAGE gels and allowed to run at 90V and resolve until the 75kDa molecular weight ladder. Next, the gels were transferred on nitrocellulose membrane at 0.2 A for 2.5 h and 4°C. Membranes were blocked with 5% skim milk for 1 h and probed with primary antibodies overnight. Primary antibodies used were: mouse anti-APOB (Santa Cruz Biotechnology, USA, Cat #13538, dilution 1:500), rabbit anti-calnexin (Sigma-Aldrich, USA, Cat #4731, dilution 1:5000), MMP2 (Abcam,UK, Cat # ab92536, dilution 1:2000), TIMP1 (Abcam,UK, Cat # ab81282, dilution 1:2000), TIMP2 (Abcam,UK, Cat #ab180630, dilution 1:2000), α-SMA (Abcam,UK, Cat #5694, dilution 1μg/mL). Blots were washed with Tris-buffered saline −0.2% Tween 20 (TBST) solution twice for 5 min each. Next, membranes were probed with the appropriate horseradish peroxidase (HRP)-conjugated secondary antibody (dilution 1:2000) for 1 h followed by three washes with TBST for 10 min each, and developed using ECL high sensitivity substrate (Immobilon Western Chemiluminescent HRP Substrate, Merck Millipore). Blots were visualized using ChemiDoc apparatus and quantified using BioRad ImageLab software.

#### RNA-seq analysis

HepG2 + LX-2 spheroids were generated for 96 h with downregulation of endogenous *IL32* using siRNA as described in the above section. Total RNA was extracted with the RNeasy Plus mini kit (Qiagen). RNA integrity number and concentration was evaluated by Agilent Bioanalyzer 2100 system (Agilent Technologies) and all samples had a RIN>6.8 and concentration >20 ng/μL. Illumina sequencing and subsequent analysis was carried out by Novogene (UK) Company Ltd., in Cambridge. Reference genome and gene model annotation files (Homo sapiens GRCh38/hg38) were downloaded from a genome website browser (NCBI/UCSC/Ensembl) directly. Indexes of the reference genome was built using STAR and 46 paired-end clean reads were aligned to the reference genome using STAR (v2.5). HTSeq v0.6.1 was used to count the read numbers mapped of each gene. And then FPKM of each gene was calculated based on the length of the gene and reads count mapped to this gene. *IL32* transcripts were grouped and reconducted to isoforms described in the literature.^64^ Differential expression analysis between two conditions/groups (three biological replicates per condition) was performed using the DESeq2 R package (2_1.6.3). The resulting p-values were adjusted using the Benjamini and Hochberg’s approach for controlling the False Discovery Rate (FDR). Genes with an adjusted p-value <0.05 found by DESeq2 were assigned as differentially expressed. For liver samples from individuals from the MAFALDA cohort, total RNA for 264 liver samples was isolated using miRNeasy Advanced Mini kit (Qiagen). RNA sequencing and library preparation was performed in a paired-end 150 bp mode using the Illumina NovaSeq PE150 (Novogene, China). Following the reads quality check (FastQC software, Babraham Bioinformatics, Cambridge, UK) and the trimming of low-quality reads and potential contaminating adapters by Trimmomatic^54^ (v0.39), reads were aligned to GRCh38 reference genome by STAR^53^ (v2.7.10a). Gene-level read counts were quantified by RSEM^52^ (v1.3.3) software against the Ensembl (release 107). Samples with insufficient mapping specificity (those with a uniquely to total mapped reads <0.7) were excluded before the analysis. Gene counts were normalized using DESeq2^51^ (v.1.38.3). The association between IL32 rs76580947 and rank-based inverse normal transformed counts was examined using a linear regression analysis adjusting for age, gender, percentage of coding bases (QC measure from Picard toolkit), RNA Integrity Number (RIN), and 5 surrogate variables detected by surrogate variable analysis (SVA).^32^

#### Plasma proteome analysis

Proteomic profiling on blood plasma samples of 54,273 UK Biobank participants was performed using the Olink Explore 1536 platform. A total of 1,463 distinct proteins were measured across four Olink panels: Cardiometabolic, Inflammation, Neurology, and Oncology. Extensive quality control steps, and calculation of Normalized Protein eXpression (NPX) values for each protein were performed internally at Olink’s facilities.^65^ We tested the association between IL32 rs76580947 and 1,463 unique protein levels in 365,495 Europeans from UK Biobank under an additive genetic model by linear regression analysis adjusting for age, gender, BMI, first 10 genomic principal components and array batch. All plasma protein values were rank-based inverse normal transformed before the analysis. We corrected the marginal associations for multiple testing using Benjamini-Hochberg method, and plasma proteins with a corrected p-value <0.05 were deemed as significant.

#### Quantification of IL32 and PLA2G2A by ELISA

In the Liver-BIBLE 2020 cohort, circulating levels of IL32 were quantified using Human IL32 DuoSet ELISA kit (DY3040, R&D Systems, Minneapolis, USA) following the manufacturer’s instructions. The assay is designed to detect IL32α, IL32β and IL32γ with a detection range of 78.5–5000 pg/mL. Plasma Samples were diluted 1:2-1:20 in PBS and were measured in duplicate. Optical density was measured at 450nm using TECAN infinite F200 PRO instrument (Männedorf, Switzerland). The minimal detectable concentration above blank was 10 pg/mL, and all samples measured at lower levels were set at 10 pg/mL. The intra-assay coefficients of variation (CV) were 3.9 ± 5.0%. PLA2G2A was measured in cell culture supernatant of primary human hepatocyte spheroids by Human PLA2G2A ELISA kit (Thermo fisher scientific) following the manufacturer’s instructions. *PLA2G2A* encodes for secretory phospholipase A2, an extracellular enzyme. Briefly, 100μL of media was collected from 10 wells that contain spheroids, and pooled together for each technical replicate. Samples were measured in triplicates. Undiluted media was measured, and the detectable concentration range was found to be high. Thus, the samples were diluted two times with PBS and the kit-provided-standards were used to plot the standard curve every time the ELISA was performed.

#### Immunofluorescent staining for type I collagen

Type I collagen (COL1A1) stained area was quantified as described before^.56^ Briefly, 8μm sections of the primary human spheroids composed of primary human hepatocytes and primary human hepatic stellate cells were acquired as described in the previous section. Then, the sections were incubated with 5% w/v Bovine Serum Albumin (BSA, Sigma-Aldrich) in PBS solution for 1 h. Rabbit anti-COLLAGEN I (Sigma-Aldrich, HPA011795) (1:100) was diluted in 5% BSA in PBS solution and incubated overnight at 4°C, followed by two washing steps of 5 min each. Next, sections were incubated with donkey anti-rabbit secondary antibody with fluorescent dye Alexa Fluor 594 (Invitrogen, dilution 1:2000) for 1 h at room temperature followed by three washes with PBS, 5 min each. Nuclei were stained by DAPI (Sigma-Aldrich, dilution 1:8000 in PBS) for 5 min. Finally, cells were mounted with fluorescence mounting medium (Dako). Fluorescent pictures were obtained using Nikon microscope with software NIS-Element 5.30.04 (Bergman Labora, Gothenburg, Sweden) Image analysis was performed using an in-house macro in ImageJ (v.1.52h, NIH) where nuclei were counted, the total spheroid area was determined, and a static threshold was applied to all images for each of the fluorescent channels to determine positively stained area.

#### Lipidomic sample preparation and analysis

Spheroids composed of primary human hepatocytes transfected with negative control scramble or *IL32* siRNA, were generated as described in the previous section, for a total of 7 days. Each sample consisted of 96 spheroids (1 plate) that were pooled into microfuge tubes. Triplicate samples were collected for each group, and representative cell numbers were counted for normalization. Lipidomic analysis was performed at Swedish Metabolomics Center, (SMC) Umeå, Sweden. At SMC, sample preparation was performed according to Folch et al. with some smaller modifications.^66^ In detail, 250 μL of extraction buffer (2:1 v/v chloroform:methanol) including internal standards and 50 μL of 0.15 M NaCl were added to 10 mg of sample material. The sample was shaken with a tungsten bead at 30 Hz for 2 min in a mixer mill, the bead was removed, and proteins were precipitated at RT for 1 h. The sample was centrifuged at +4°C, 14 000 rpm, for 3 min. 160 μL of the lower chloroform phase were transferred to a micro vial and stored at −80°C until LC/MS analysis. The chromatographic separation was performed on an Agilent 1290 Infinity UHPLC-system (Agilent Technologies, Waldbronn, Germany). 0.5–1 μL of extracted plasma or tissue sample were injected onto an Acquity UPLC CSH, 2.1 × 50 mm, 1.7 μm C18 column in combination with a 2.1 mm × 5 mm, 1.7 μm VanGuard precolumn (Waters Corporation, Milford, MA, USA) held at 60°C. The gradient elution buffers were A (60:40 acetonitrile:water, 10 mM ammonium formate, 0.1% formic acid) and B (89.1:10.5:0.4 2-propanol:acetonitrile:water, 10 mM ammonium formate, 0.1% formic acid), and the flow-rate was 0.5 mL per min. The compounds were eluted with a linear gradient using initial condition 15% B, and increase to 30% B at 1.2 min, 55% at 1.5 min, isocratic to 5.0 min, increase to 72% B at 7 min, 85% B at 9.5 min and 100% B at 10.0 min, and then held at 100% for 2 min. An additional wash of the injection valve, with 100% B and flowrate 4.0 mL min-1 for 0.3 min, was performed before decreased to initial condition 15% B over 0.3 min flow rate 0.5mL per min; these conditions were held for 1.1 min to equilibrate the column before next injection.

The compounds were detected with an Agilent 6546 Q-TOF mass spectrometer equipped with an electrospray ion source operating in positive ion mode. A reference interface was connected for accurate mass measurements; the reference ions purine (4 μM) and HP-0921 (Hexakis(1H, 1H, 3H-tetrafluoropropoxy) phosphazine) (1 μM) were infused directly into the MS at a flow rate of 0.05 mL min-1 for internal calibration, and the monitored ions were purine m/z 121.05; HP-0921 m/z 922.0098 for positive. The gas temperature was set to 150°C, the drying gas flow to 8 L per min and the nebulizer pressure 35 psig. The sheath gas temp was set to 350°C and the sheath gas flow 11 L per min. The capillary voltage was set to 4000 V in positive ion mode. The nozzle voltage was 300 V. The fragmentor voltage was 120 V, the skimmer 65 V and the OCT 1 RF Vpp 750 V. The collision energy was set to 0 V. The m/z range was 70–1700, and data was collected in centroid mode with an acquisition rate of 4 scans s-1 (1977 transients/spectrum). Using Batch Targeted Feature Extraction on all samples, the peak areas of the lipid features, as well as the internal standards, were calculated in Agilent MassHunter Profinder version B.10.00 (Agilent Technologies Inc., Santa Clara, CA, USA). In-House libraries with exact masses and retention times were used for tentative annotation of lipids. All multivariate statistical investigations (PCA, OPLS-DA) were performed using the software package SIMCA version 17.0 (Sartorius AG, Umeå, Sweden).

### Quantification and statistical analysis

Statistics were performed using GraphPad Prism version 9 for *in vitro* experiments. For genetic studies, no randomization was performed for individuals. For *in vitro* studies, primary and immortalized cells have been allocated into study groups randomly. For all analysis, power calculation was not performed to estimate sample sizes and investigators were not blinded during experimental analysis. For *in vitro* experiments with immortalized cells the number of experiments performed is calculated based on a) the effect size observed after three experiments and b) based on the presence of experiments that gave results classified as outliers based on the function “Identify Outliers” from the GraphPad prism statistics software. For *in vitro* experiments with primary cells the number of experiments is based on the availability of cryopreserved cells. The mean value of all the experiments is then pooled together and analyzed. Sample size (n) values are provided in the figures and statistical tests performed are described in the figure legends. All data are presented as Mean and standard deviation and is indicated in figure legends. For cell lines and primary cells, “N” represents one independent experiment performed with separate media and reagents. Each independent experiment consists of greater than 3 technical replicates, such as for imaging, ELISA, AdipoRed staining. Similarly, for transcriptomics and lipidomics, several organoids (technical replicates) are pooled for one independent experiment.

For *in vitro* experiments, statistical differences among groups were calculated with parametric t test, nonparametric Mann-Whitney U tests. and one-way analysis of variance (ANOVA). p values < 0.05 were considered as statistically significant. All the reported p-values are two sided. The association between alanine aminotransferase (ALT) and 194 common imputed variants (MAF ≥0.01, imputation INFO score ≥0.8) of IL32 gene (±50 kb flanking region) in European subset (N = 425,671) of UK Biobank (UKBB) was performed using a linear mixed-effects as implemented in BOLT-LMM, version 2.3.4.^29^^,^^67^ The association with other continuous (glucose, cholesterol, high-density lipoprotein cholesterol, low-density lipoprotein cholesterol, and triglycerides) and categorical traits (chronic liver disease, liver cirrhosis and severe liver disease) was examined using a linear or logistic regression analysis in a subset of unrelated Europeans (after excluding 3rd degree or closer relatives from the European subset, N = 365,495), respectively.^49^ An additive genetic model was assumed for all analyses; continuous traits were rank-based inverse normal transformed prior to the analysis, with all models adjusted for age, gender, body mass index (BMI), the first 10 principal components of ancestry, and genotyping array.

To extract proton density fat fraction (PDFF) in UKBB, we used 44,555 neck-to-knee body MRI images from Dixon technique (data field 20201) using a deep learning approach as explained before.^68^ Briefly, we used a total of 9,893 PDFF values (data field 22436) as the reference (ground truth) data to train the ResNet-50 convolutional neural network (CNN) in PyTorch (version 1.10).^69^ Reference PDFF dataset was split into training (70%, n = 6,897) and validation (30%, n = 2,961) sets, and both coefficient of determination (R^2^ = 0.963) and mean absolute error (MAE = 0.632) on the validation set outperformed the previously trained model on a similar but smaller dataset.^68^

The association between the *IL32* rs76580947 and liver steatosis (steatosis absence and mild versus severe) within 3 cohorts (i.e., Finnish, southern and central Italy) was evaluated under an additive genetic model by a binary logistic regression analysis adjusted for age, gender, BMI and recruitment center (for the Finnish cohort) using MATLAB R2021a (MathWorks). All individuals with a missing steatosis diagnosis were excluded prior to the analysis. The estimates were then combined using a fixed-effect the inverse-variance weighted meta-analysis by using “meta” package (http://cran.r-project.org/web/packages/meta/index.html) in R, version 3.6.1. Similarly, we meta-analysed the summary statistics of IL32 rrs76580947 with SLD in four independent European studies (Figure S4) The impact of *IL32* rs76580947 variant on the risk of having high circulating IL32 was examined in the Liver-BIBLE cohort 2020, by multivariable logistic regression analysis, with elevated IL32 levels as the outcome (falling in the 4^th^ vs. other quartiles), adjusted for age, gender, BMI and analysis batch. Participants were stratified by quartiles of circulating IL32 due to: i) nonnormal distribution; ii) wide range of variability; iii) approximately one-quarter of individuals having undetectable-low circulating levels; iv) high variability at the upper end of distribution.

## Supplemental information

Document S1. Figures S1–S4 and Tables S1–S6

Document S2. Article plus supplemental information
