# Supplementary material for: IL32 downregulation lowers triglycerides and type I collagen in di-lineage human primary liver organoids
Source: Cell Rep Med. 2024 Jan 16;5(1):101352. doi: 10.1016/j.xcrm.2023.101352 (PMC10829727; doi:10.1016/j.xcrm.2023.101352)
Supplement: Document S1. Figures S1–S4 and Tables S1–S6 [file mmc1.pdf]

Cell Reports Medicine, Volume *5*

Supplemental information

IL32 downregulation lowers triglycerides and type I collagen in di-lineage human primary liver organoids

Kavitha Sasidharan, Andrea Caddeo, Oveis Jamialahmadi, Francesca Rita Noto, Melissa Tomasi, Francesco Malvestiti, Ester Ciociola, Federica Tavaglione, Rosellina M. Mancina, Alessandro Cherubini, Cristiana Bianco, Angela Mirarchi, Ville Männistö, Jussi Pihlajamäki, Vesa Kärjä, Stefania Grimaudo, Panu K. Luukkonen, Sami Qadri, Hannele Yki-Järvinen, Salvatore Petta, Silvia Manfrini, Umberto Vespasiani-Gentilucci, Vincenzo Bruni, Luca Valenti, and Stefano Romeo

Supplementary information

Supplementary figures

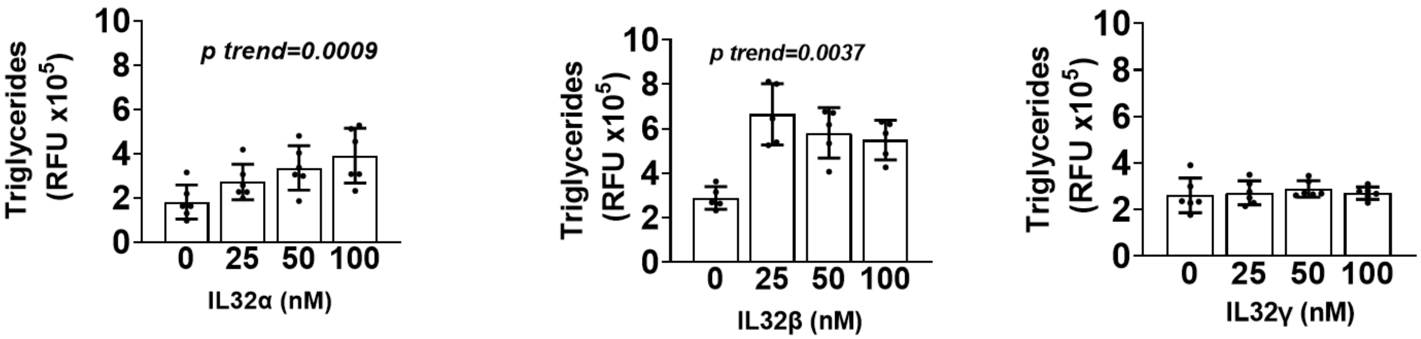

Supplementary Figure 1: Dose response relationship of IL32 isoforms and intracellular triglycerides. Related to figures 1 and 2.

HepG2 + LX-2 cells were cultured as spheroids for a total of 96 hours. Initially, 48 hours after seeding cells the media was supplemented with different concentrations of human recombinant IL32α, IL32β or IL32γ for a total of 96 hours. Intracellular neutral fat content measured by AdipoRed assay.

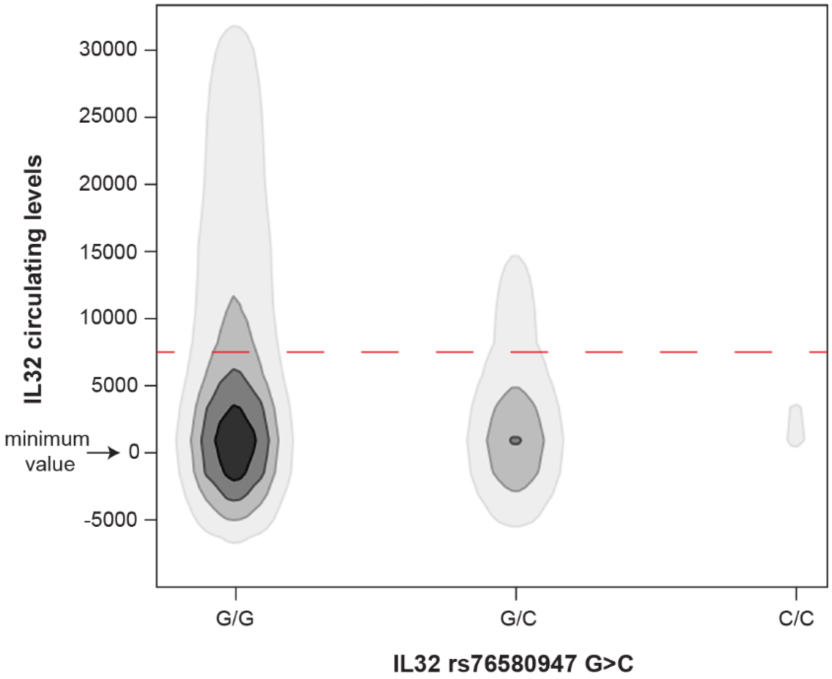

Supplementary Figure 2. IL32 circulating levels distribution in Liver-BIBLE 2020 cohort. Related to Table 2. Density contour plot showing the distribution of IL32 circulating levels in participants stratified by IL32 rs76580947 genotype. The red dotted line represenst the treshold of 4th quartile.

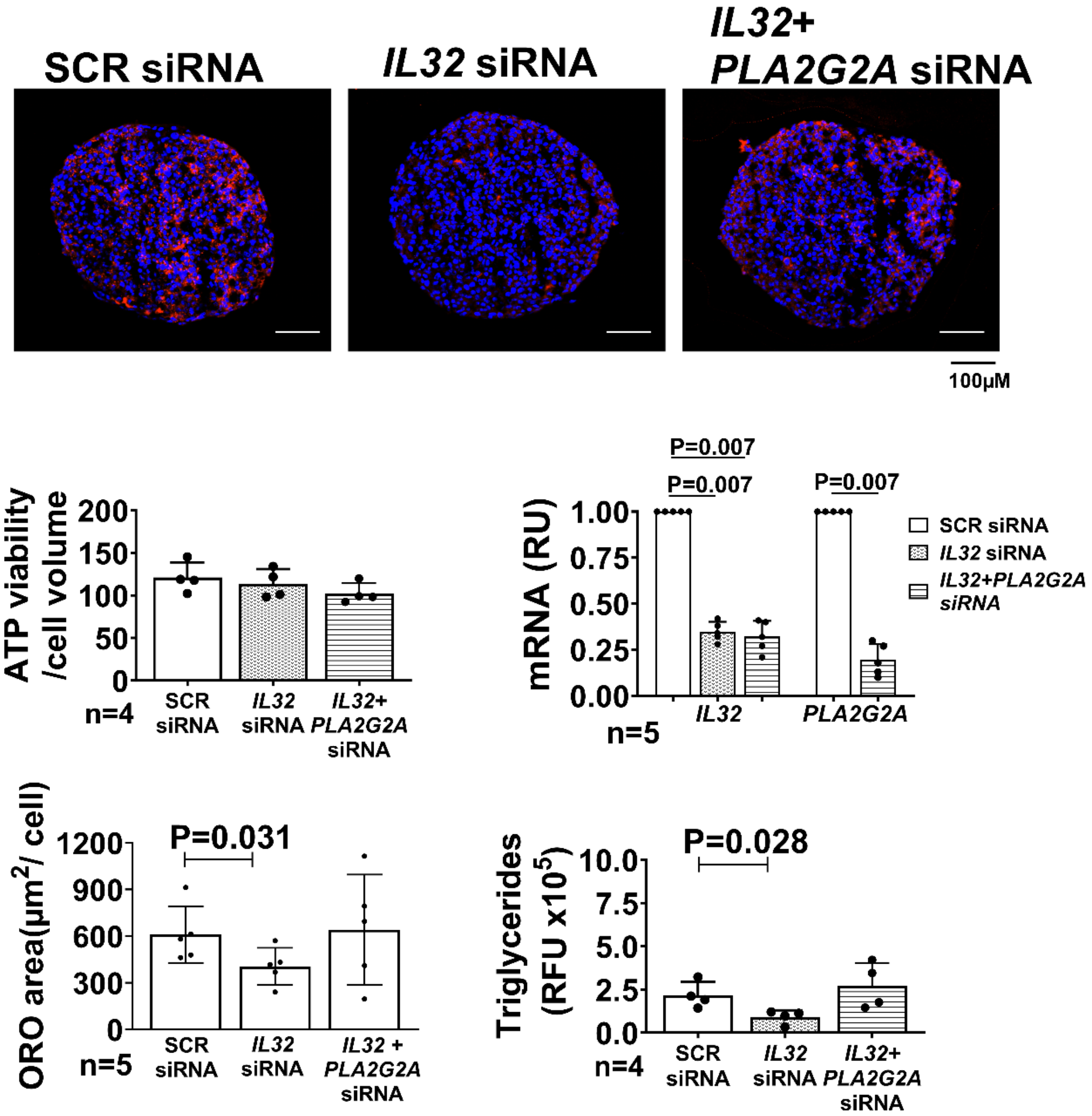

Supplementary Figure 3. **Co-downregulation of *PLA2G2A* and *IL32* abolished the IL32-regulated loss in intracellular triglycerides in HepG2+LX-2 spheroids. Related to Figure 6.**
Endogenous *IL32* and *PLA2G2A* were downregulated individually or in combination using siRNA in spheroids for a total of 96 hours in. Cellular ATP levels (marker of viability) remained unchanged between the three groups and there was a 75-80% reduction in mRNA levels of *IL32* and *PLA2G2A*, relative to beta actin. Intracellular neutral fat content measured by Oil Red-O staining, normalized to DAPI stained nuclei demonstrated decreased intracellular triglycerides content after *IL32* downregulation while the co-downregulation of *PLA2G2A* and *IL32* abolished this effect. Data shown as mean ±SD for the reported number of experiments

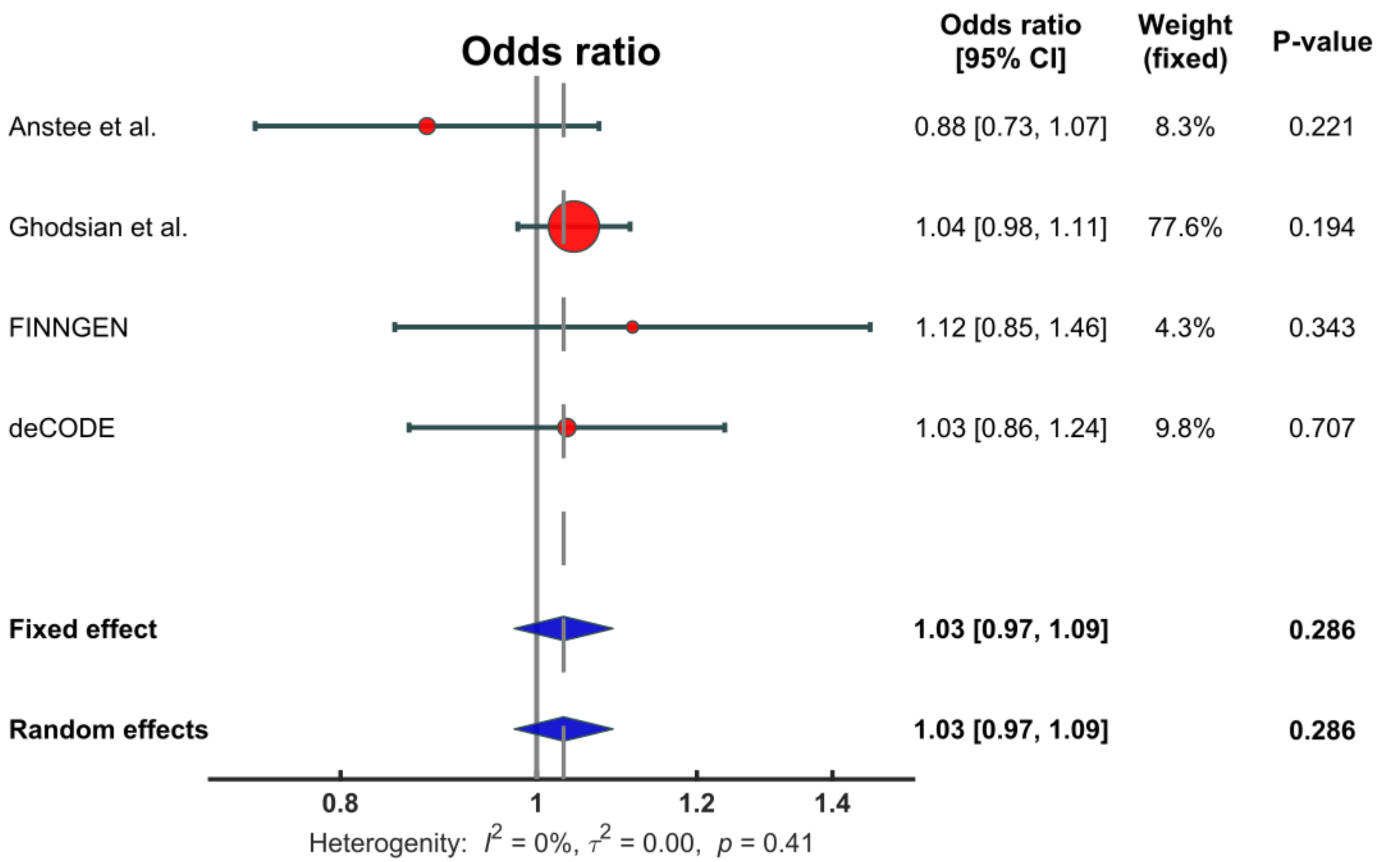

Supplementary figure 4: Meta-analysis for IL32 rs rs76580947 with SLD in four independent European studies. Related to figure 7C.

Pooled effect estimate was calculated using inverse-variance–weighted fixed- and random-effects meta-analysis. I2, τ2 (between-study variance) and p-value for Cochran's Q heterogeneity test have been reported to assess the between-study heterogeneity.

Supplementary tables:

**Phenotype**	**N**	**95% CI**	***Beta/OR***	***P-value***
Age	365,495		0.007	0.07
BMI	364,297		-0.001	0.72
ALT	414,214		-0.02	9.90×10^{-13}
AST	347,125		-0.02	3.2×10^{-10}
Glucose	318,661		0.004	0.31
Cholesterol	348,409		-0.01	0.009
HDL-C	318,896		0.001	0.78
LDL-C	347,760		-0.01	0.012
Triglycerides	348,128		-0.006	0.10
PDFF	35,521		-0.002	0.86
Gender, male, n%	168,988(46.2)	0.98- 1	1	0.93
Chronic liver disease, n%	9,212(2.52)	0.92- 1	0.97	0.34
Cirrhosis, n%	2,457(0.673)	0.83- 1	0.91	0.10
Severe liver disease, n%	1,249(0.417)	0.71- 0.96	0.82	0.013
Hepatocellular carcinoma, n%	325(0.0891)	0.60- 1.1	0.81	0.17
Diabetes mellitus, n%	25,765(7.05)	0.97- 1	1	0.99

Supplementary table 1. Characteristics of UK Biobank individuals with IL32 rs76580947. **Related to Figure 7A.** Continuous traits are shown as mean and standard deviation or median (interquartile range) as appropriate. Gender is shown as number and proportion. The association of ALT with IL32 rs76580947 was calculated using a linear mixed-effects model, and linear/logistic regression model for other traits. All analyses were adjusted for age, gender, BMI, the first 10 principal components of ancestry, and genotyping array. Continuous traits were rank-based inverse normal transformed before regression analyses. Severe liver disease is defined as a composite diagnosis of cirrhosis, decompensated liver disease, hepatocellular carcinoma, and/or liver transplantation of 22,812 UK Biobank participants of European descent without clinical history of liver disease and liver cancer from the National Health Service records[50]

Phenotype	95% CI	OR	*P value*
Age, Years	0.97-1.01	0.99	0.42
Gender, F	0.57-1.32	0.87	0.51
IL32 rs76580947 G>C	0.46-0.94	0.66	0.012
BMI Kg/m^2	0.95-1.05	1.00	0.93

	**Overall**	**GG**	**GC**	**CC**	**P**
**N=**	955	745 (78.0%)	193 (20.2%)	17 (1.8%)	0.28
Age, Years	53,9±6.4	53.9±6.4	53.6±6.1	58.0±6.1	0.35
Gender, Female	158(16.5%)	139 (18.6%)	16 (8.3%)	3 (17.6%)	0.004
BMI, Kg/m2	28.5±3.1	28.5±3.1	28.7±2.9	28.8±3.1	0.47
Diabetes, Yes	35 (3.7%)	29 (3.9%)	6 (3.1%)	0	0.39

Supplementary table 2. Independent predictors of high circulating IL32 levels (top table) and Clinical features of the Liver-Bible 2020 cohort (bottom). Circulating IL32 defined as in the highest quartile of its distribution in the Liver-Bible cohort. Related to figure 7C. The association was tested by nominal logistic regression under an additive genetic model adjusted by age, gender, BMI and analysis batch. The association for clinical features was tested by generalized linear models (age and BMI) or nominal logistic regression (gender and diabetes).
BMI: body mass index.

Protein (gene)	N	A2	A1	A1 Freq	Beta	P-value	95% CI	SE
Interleukin 32 (IL32)	37808	G	C	0.08	-0.04	1.8×10^{-244}	-0.44, -0.39	0.01
Chymotrypsinogen B (CTRB1)	37869	G	C	0.08	-0.05	6.8×10^{-6}	-0.08, -0.03	0.01
Trypsin-2 (PRSS2)	38008	G	C	0.08	-0.05	1.9×10^{-5}	-0.08, -0.03	0.01
All-trans-retinol dehydrogenase [NAD(+)] (ADH4)	37198	G	C	0.08	-0.04	2.5×10^{-5}	-0,07.-0.02	0.01
Carboxypeptidase B (CPB1)	38327	G	C	0.08	-0.05	2.9×10^{-5}	-0,07.-0.02	0.01
Glutathione S-transferase A1 (GSTA1)	37842	G	C	0.08	-0.04	1.1×10^{-4}	-0.06, -0.02	0.01
Glutathione S-transferase A3 (GSTA3)	36980	G	C	0.08	-0.04	1.9×10^{-4}	-0.06,-0.02	0.01
Sorbitol dehydrogenase (SORD)	38001	G	C	0.08	-0.04	2.4×10^{-4}	-0.06,-0.02	0.01
Neural cell adhesion molecule 1 (NCAM1)	38008	G	C	0.08	-0.04	2.7×10^{-4}	-0.06,-0.02	0.01

Supplementary Table 3: The association between IL32 rs76580947 and 1,463 unique protein levels in 365,495 Europeans from UK Biobank under an additive genetic model by linear regression analysis adjusting for age, gender, BMI, first 10 genomic principal components and array batch. Related to Figure 7C.

All plasma protein values were rank-based inverse normal transformed before the analysis. Only plasma proteins with a corrected P-value< 0.05 are shown.

**Steatosis severity**	**P**	**OR**	**CI**	
South Italy	0.53	0.87	0.56	1.34
Central Italy	0.93	0.98	0.61	1.59
Finnish	0.38	0.76	0.41	1.40
Meta analyses				
Fixed-effect	0.38	0.88	0.66	1.17

**Fibrosis severity**				
South Italy	0.15	1.35	0.90	2.00
Central Italy	0.24	1.35	0.83	2.20
Finnish	0.80	0.93	0.52	1.68
Meta analyses				
Fixed-effect	0.13	1.24	0.95	1.64

**Inflammation severity**				
South Italy	0.22	1.34	0.84	2.14
Central Italy	0.59	1.16	0.67	2.01
Finnish	0.58	0.81	0.38	1.71
Meta analyses				
Fixed-effect	0.37	1.16	0.84	1.60

**Ballooning severity**				
South Italy	0.51	1.16	0.75	1.82
Central Italy	0.36	1.29	0.76	2.21
Finnish	0.54	0.75	0.29	1.90
Meta analyses				
Fixed-effect	0.41	1.14	0.83	1.58

**Steatosis presence***	**P**	**OR**	**CI**	
Central Italy	0.78	1.09	0.58	2.04
Finnish	0.46	0.79	0.40	1.50
Meta analyses				

Fixed-effect	0.77	0.94	0.60	1.46

**Fibrosis presence**				
South Italy	0.25	1.50	0.75	3.04
Central Italy	0.59	1.18	0.65	2.17
Finnish	0.87	0.96	0.53	1.74
Meta analyses				
Fixed-effect	0.41	1.17	0.81	1.67

**Inflammation presence**				
South Italy	0.79	1.19	0.34	4.13
Central Italy	0.53	1.20	0.68	2.15
Finnish	0.66	0.85	0.40	1.79
Meta analyses				
Fixed-effect	0.76	1.07	0.70	1.65

**Ballooning presence**				
South Italy	0.72	1.12	0.61	2.06
Central Italy	0.17	0.56	0.24	1.30
Finnish	0.55	0.75	0.30	1.93
Meta analyses				
Fixed-effect	0.47	0.86	0.55	1.32

Supplementary table 4: **Meta-analyses of the association between the rs76580947 and liver histological traits in three independent cohorts: South Italy, central Italy and Finnish. Related to Fig 7C.** The association was tested by binary logistic (disease presence) or ordinal regression (disease severity) analysis under an additive genetic model adjusted by age, gender, BMI, centre of recruitment (for finnish cohort).*analyses performed only in central Italy and Finnish cohort

Phenotype	N	A2	A1	A1 Freq	Beta	P-Value	95% CI	SE
FIB4	337718	G	C	0.08	-0.004	0.27	-0.01, -0.003	0.003
FNI score	302272	G	C	0.08	-0.018	6.8×10^{-6}	-0.02, -0.009	0.003
Fatty liver index	346679	G	C	0.08	-0.004	0.04	-0.007, -0.0001	0.001
NFS	309073	G	C	0.08	0.005	0.177	-0.002, 0.013	0.003
APRI	337724	G	C	0.08	-0.019	1.2×10^{-5}	-0,027, -0.01	0.004
Apolipoprotein B	345561	G	C	0.08	-0.010	0.01	-0.01, -0.001	0.004

Supplementary Table 5. The association between IL32 rs76580947 and clinical liver fibrosis scores and apolipoprotein B levels in 365,495 European participants from UK Biobank. Related to Figure 7E.

The analysis was performed under additive model and using linear regression adjusting for age, gender, BMI, first 10 genomic principal components and array batch. All traits were rank-based inverse normal transformed prior to the analysis.

	**Race**	**Age**	**BMI**	**Cause of death**	**Medical history**
BGW-M00995 Human hepatocytes (male)	Caucasian	50	20.4	Cardiovascular accident	Hypertension- 8-9 years
BGF-M00995 Human hepatocytes (male)	Hispanic	56	30.6	Anoxia	Type 2 Diabetes x 5 yrs; Hypertension, Hyperlipidemia and Hypercholesterolemia x 1 yr; Anxiety/Depression, Arthritis, Osteoporosis, Ventricular fibrillation - all unknown duration. COVID negative.
TFE-S00354- Human hepatic stellate cells (male)	Caucasian	50	20.4	Cardiovascular accident	Hypertension- 8-9 years
NGU-S00354- human hepatic stellate cells	Caucasian	46	32.1	Other	none

Supplementary Table 6. Primary human hepatocyte and hepatic stellate cell characteristics. Related to figures 2 and 3.
